# Lights and Shadows: A Comprehensive Survey on Cooperative and Precoding Schemes to Overcome LOS Blockage and Interference in Indoor VLC

**DOI:** 10.3390/s21030861

**Published:** 2021-01-28

**Authors:** Máximo Morales Céspedes, Borja Genovés Guzmán, Víctor P. Gil Jiménez

**Affiliations:** 1 Department of Signal Theory and Communications, University Carlos III of Madrid, Leganés, 28911 Madrid, Spain; maximo@tsc.uc3m.es; 2 IMDEA Networks Institute, Leganés, 28918 Madrid, Spain; borja.genoves@imdea.org

**Keywords:** VLC, indoor, cooperative schemes, precoding, LoS blockage

## Abstract

Visible light communications (VLC) have received significant attention as a way of moving part of the saturated indoor wireless traffic to the wide and unregulated visible optical spectrum. Nowadays, VLC are considered as a suitable technology, for several applications such as high-rate data transmission, supporting internet of things communications or positioning. The signal processing originally derived from radio-frequency (RF) systems such as cooperative or precoding schemes can be applied to VLC. However, its implementation is not straightforward. Furthermore, unlike RF transmission, VLC present a predominant line-of-sight link, although a weak non-LoS component may appear due to the reflection of the light on walls, floor, ceiling and nearby objects. Blocking effects may compromise the performance of the aforementioned transmission schemes. There exist several surveys in the literature focused on VLC and its applications, but the management of the shadowing and interference in VLC requires a comprehensive study. To fill this gap, this work introduces the implementation of cooperative and precoding schemes to VLC, while remarking their benefits and drawbacks for overcoming the shadowing effects. After that, the combination of both cooperative and precoding schemes is analyzed as a way of providing resilient VLC networks. Finally, we propose several open issues that the cooperative and precoding schemes must face in order to provide satisfactory VLC performance in indoor scenarios.

## 1. Introduction

Visible light communications (VLC) are now envisaged as a mature and promising technology for future wireless communications [1,2,3]. It has been proposed for many different scenarios, both indoors and outdoors [4]. For indoor applications, there exists a wide variety of applications, such as extending the capacity and complementing the radio frequency (RF) systems [5], positioning and resource monitoring [6,7], intruder detection [8] or providing coverage where RF transmission is not allowed [9], among others. Outdoor VLC applications comprise vehicle-to-vehicle communications [10,11,12] or some trials and analysis for long distance transmission [13,14,15]. In this context, VLC have been standardized as 802.15.7 [16], and considering their integration in the IP network, usually referred to as light fidelity (LiFi), under the standard 802.11 bb [17].

In order to ease the reading of the paper, the list of acronyms has been summarized in Table 1, whereas the list of variables and symbols is in Table 2.

Since VLC make use of the optical frequency range between 400 and 800 THz, a large bandwidth is available for data transmission. The use of these frequencies may enable new wireless services that the traditional RF systems can no longer satisfy due to the imminent RF spectrum crunch. Furthermore, the optical frequency range is unregulated, which eases the research, development and deployment of VLC systems. VLC are complementary to the RF counterparts and thus, both of them can be used simultaneously and even as cooperative technologies [5]. Transmitters for VLC are based on off-the-shelf light emitting diodes (LED) that are present in the new lighting infrastructures and are being the replacement of previous lighting infrastructures due to their efficiency, which makes this technology low-cost and accessible everywhere. It means that any source of light can be tuned to be used as an access point (AP) to provide both illumination and now, data transmission. In this sense, each of these optical APs can be integrated in the heterogeneous cellular networks as an attocell [18,19] in order to extend the coverage and increase the achievable throughput of the overall system. On the receiver side, any device with photo-sensing capabilities may be used, such as photodiodes (PDs) [20], solar cells [21], image sensors, i.e., cameras [22], or even LEDs [23]. It is worth noticing that VLC also hold the belief that they are healthier than RF transmission because light does not affect the human body [24] as long as it complies with the illumination regulation. Since light does not go through solid elements, it also confers an inherent security umbrella upon VLC [25], which is a very appreciated property nowadays, where security and safety awareness are growing up in our society. However, the obstructions in the direct link between transmitter and receiver for VLC are critical for communication and it is one of its main disadvantages, which is known as the line-of-sight (LoS) link blockage or shadowing [26]. Links are often obstructed in normal scenarios due to either human body, furniture or other elements. If the system must provide a high data rate and reliable transmission network, this issue must be carefully addressed [27].

**Figure 1:**
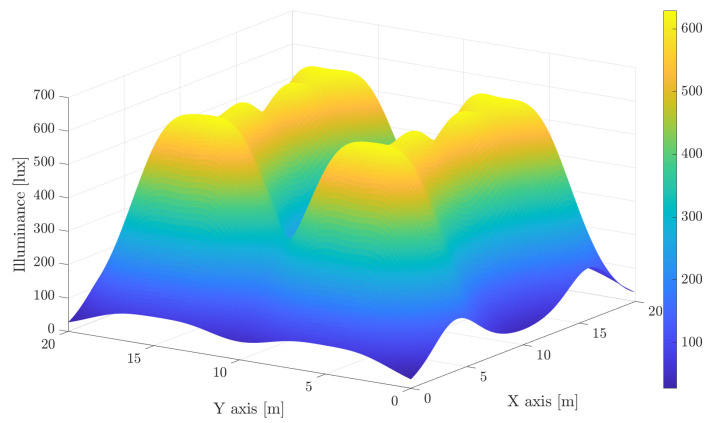
Illuminance in an indoor scenario. Beam angle of $20^{\circ }$.

**Figure 2:**
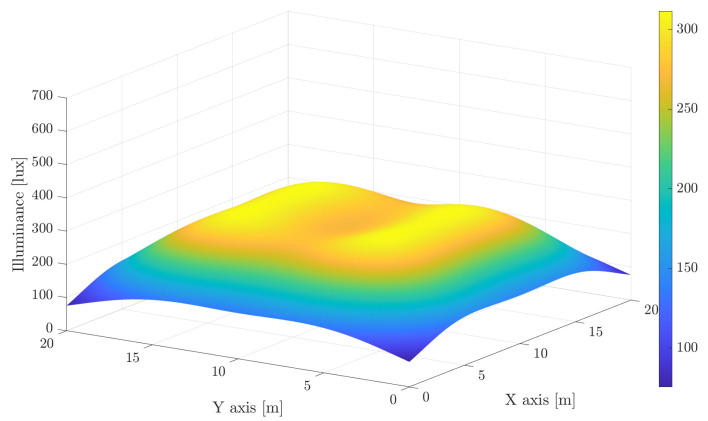
Illuminance in an indoor scenario. Beam angle of $50^{\circ }$.

Providing proper lighting within an area according to the illumination standards entails necessarily the intrinsic overlapping of light beams in order to avoid non-illuminated regions. Although these overlapped areas suffer from high interference, it can be intelligently handled to provide a reliable communication without outages. For illustrative purposes, let us consider a 20m×20m×7m scenario, which may correspond to an industrial building. In this scenario, a set of luminaries (Specifically, we have considered the LEDs Citizen CLU058-3618C4 [28], which are mainly intended for illumination. A discussion about the LEDs employed for high data rate VLC is provided afterwards.) providing about 24,000 lm each has been deployed on the ceiling. The obtained illuminance assuming a concentrator lens at each LED that generates a beam angle of $20^{\circ }$ and $50^{\circ }$ is shown in Figure 1 and Figure 2, respectively, assuming a receiving plane 1.5 m above the floor. For comparison purposes, both figures consider the same range for the illuminance index. Notice that the first trade-off is related to providing a constant and satisfactory illuminance. Narrow beams obtain peaks of illuminance above 500 lux, while shadowing below 100 lux may appear in some areas. However, as seen in Figure 2, using wider beams involves reducing the average illuminance to about 250 lux. On the other hand, increasing the number of LED lights also increases complexity, cost and interference.

**Figure 3:**
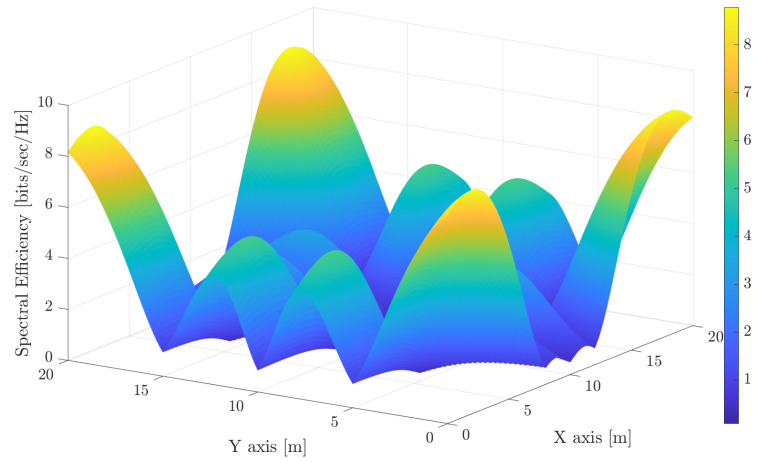
Spectral efficiency without cooperation among transmitters. Beam angle of $20^{\circ }$.

**Figure 4:**
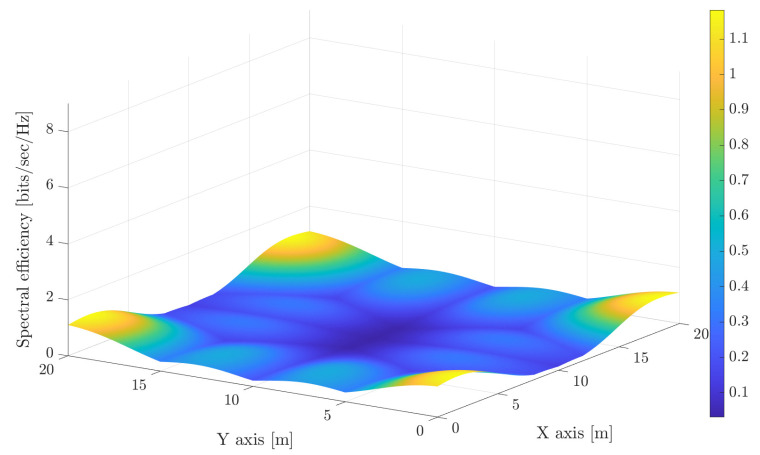
Spectral efficiency without cooperation among transmitters. Beam angle of $50^{\circ }$.

Moving to VLC, the second trade-off is related to the interference. In this sense, it is obvious that avoiding non-illuminated areas by overlapping the illuminance footprint of the luminaries involves increasing the interference. The achievable spectral efficiency (The receiver parameters are the same as in [29]. Furthermore, the noise is considered negligible so that the spectral efficiency does not depend on the modulation bandwidth.) for the considered configuration is shown in Figure 3 and Figure 4, where each LED light works as an independent AP, i.e., without any cooperation among them. As occurs in previous figures, the same range for the spectral efficiency index is considered for comparison purposes. It is shown that using narrow beams reduces the interference among optical APs, and therefore, great spectral efficiency is achieved around each optical AP. However, very low spectral efficiency is achieved in the overlapped areas. Furthermore, using wider beams, which recalls that it is required for providing a uniform illumination (see Figure 2) and leads to a poor spectral efficiency as it can be seen in Figure 4, because most of the locations of the scenario are subject to overlapping from several optical transmitters, i.e., to inter-cell interference.

At this point, the two main requirements for VLC are presented: providing satisfactory illumination and data transmission simultaneously, which involves a trade-off between overlapping and interference. This way, as the primary functionality of lighting fixtures is to provide a standard compliant illumination, overlapping areas are unavoidable and interference management techniques must be invoked. In this context, cooperation among transmitters widely analyzed for RF cellular networks [30] have recently been proposed for VLC [31]. Besides, enabling cooperation among several optical APs inherently generates a multiple-input multiple-output (MIMO) network [32], allowing us to implement the well-known precoding schemes derived for RF systems [33,34]. Both cooperation and precoding techniques can be combined in order to create clusters of optical APs from a user-centric perspective [35]. This paradigm involves VLC networks more resilient to LoS-link blocking effects. However, the step of implementing cooperation and precoding schemes originally derived for RF systems to VLC networks is not straightforward. In this sense, this work presents a comprehensive analysis and evaluation of the state of the art by considering the need for overcoming the LoS-link blockage. The organization of this work is described as follows:

- In Section 2, the system model for VLC is analyzed, presenting a brief overview of the single and multi carrier modulation schemes. The network requirements for implementing cooperative and precoding schemes are presented considering the transmitter, receiver and network architectures, which involve parameters such as dynamic range, noise, field of view (FoV) or the need for backhaul links between transmitters, among others.
- Section 3 motivates the need for employing cooperative schemes in VLC scenarios and presents the state of the art cooperative techniques for VLC published in the literature. Fundamentals of every cooperative technique are described and compared to its counterparts.
- Section 4 is devoted to introducing the concept of precoding schemes from the signal processing perspective and for analyzing their application to VLC systems. First, the fundamentals of precoding schemes and its particularities for VLC are presented. After that, the differences between the capacity for RF and VLC systems are highlighted. Specifically, for VLC, the typical Shannon capacity equation [36,37], i.e., $\log_{2}\left(1+\mathrm{SNR}\right)$, where SNR is the signal to noise ratio, does not hold, since the optical channel is amplitude-constrained. In this sense, the lower and upper bounds of the capacity for VLC derived in [38,39,40] are presented. Moreover, the capacity bounds assuming MIMO precoding in VLC systems derived in [34] are analyzed. Finally, we present an evaluation of the state of the art for precoding applied to VLC networks.
- In Section 5, a review of the intra-cluster and inter-cluster interference management in VLC networks is presented, considering the combination of cooperative and precoding schemes. After that, the concept of user-centric clustering is analyzed reviewing the recent works that apply this approach in VLC networks [35]. Furthermore, VLC systems are usually deployed in indoor scenarios, where an umbrella RF system, e.g., WiFi or femtocells, is already available. Enabling cooperation among VLC and RF systems, the concept of hybrid VLC/RF networks is introduced and the recent works in the field are analyzed.
- In Section 6, remaining challenges and open issues for cooperative and precoding schemes in VLC networks subject to shadowing are outlined. These challenges comprise adapting the transmission schemes to limited backhaul links, modeling the channel state information (CSI) prediction for users mobility, uncorrelating the channel responses among users, novel user-centric clustering schemes or the application of the massive MIMO concept to VLC.

It is worth noting that there are several surveys in the field of VLC networks, e.g., [6,11,41,42,43,44]. However, none of these articles are focused on managing the blocking and shadowing effects, which indeed is one of the main VLC limitations. With this work, our goal is to present a clear and comprehensive picture of the cooperative and precoding schemes for VLC and how they are useful for overcoming the harmful blocking and shadowing effects. They invoke techniques that exploit multiple rays, arriving at the destination, either by reflection [45] or by using different transmission points [31], the cooperation among APs [46,47], and signal processing techniques associated to reduce or manage interference [48].

## 2. Modeling the VLC Networks: Architecture, System Model and Scenarios

This section describes the architecture of VLC networks, as well as the main mathematical formulation to provide an application framework. At the end of the section, the scenarios are classified into two groups describing the main applications.

### 2.1. VLC Architecture

The architecture in VLC networks is composed of one or several transmitters and receivers. Transmitters are commonly denoted as optical APs, which are usually interconnected by a backhaul link for the interchange of information, and receivers are users randomly deployed over the scenario. Moreover, from a cellular perspective, each optical AP provides coverage creating an attocell [49].

#### 2.1.1. Transmitter

The optical APs based on LED technology are typically deployed on the ceiling of rooms, corridors or another area in order to avoid shadowed zones and to generate a uniform illumination. For high data rate VLC, the LEDs must provide a large modulation bandwidth while ensuring satisfactory lighting [50]. In this sense, there are two alternatives for generating white light illumination; using blue emitters with a phosphor layer or multicolor red-green-blue (RGB) integrated circuits so that the combination of colors results in white lighting. Although multicolor LEDs provide larger bandwidth, phosphor LEDs are widely proposed for VLC, because of their lower cost, lower complexity and illumination properties. Moreover, μLEDs are currently an alternative technology for VLC transmitters subject to providing lower illumination than phosphor LEDs [51].

The electrical equivalent of an LED is analyzed in [52]. Specifically, the modulation bandwidth depends on the bias capacitance of the LED electrical equivalent. In this sense, in [52], it is shown that the modulation of a phosphor white LED OSRAM Ostar [53] is greater than 40 MHz. This way, the illumination LEDs Citizen Citiled considered in Figure 1 and Figure 2 achieve up to 1 MHz bandwidth for industrial applications [54], which allows us to provide connectivity in hostile environments, such as the construction of tunnels, although it cannot be considered as a high data rate application. Focusing on high data rate VLC, in [55], it is shown that using equalization techniques a modulation bandwidth of up to 416 MHz can be achieved. In this context, the maximum data rates achieved by RGB LEDs, phosphor LEDs and μLEDs are equal to 15.73 Gb/s [56], 3 Gb/s [57] and 7.91 Gb/s [58], respectively. However, these rates are subject to be surpassed in the future.

**Figure 5:**
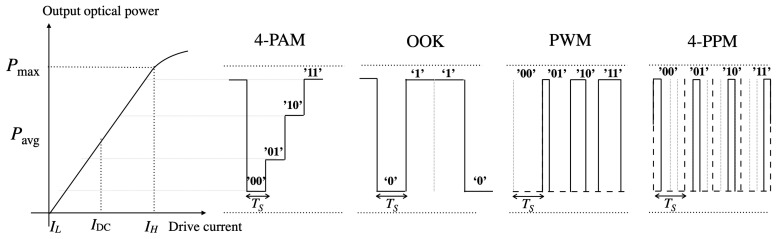
Dynamic range of the LED optical transmitter. Single carrier modulations: 4-PAM, OOK, PWM and 4-PPM. $T_{S}$ denotes the symbol period.

In contrast to RF systems, transmission in VLC is based on intensity modulation and direct detection (IM/DD). That is, the intensity of the signal at the input of the optical transmitter is modulated and the received energy is collected by a PD, which converts the optical power into an electrical signal. This condition yields the following constraint; the transmitted signal must be real and non-negative. As a consequence, imposing IM/DD involves relevant differences between VLC and RF systems, e.g., it is unfeasible to apply coherent detection to VLC systems, which is usually considered in RF transmission. As illustrated in Figure 5, optical APs provide illumination over a limited drive current range between $I_{L}$ and $I_{H}$, where $I_{L}$ is typically zero and $I_{H}$ corresponds to the saturation current. Within this range, the APs provide an optical power between zero and $P_{\max}$. Moreover, for IM/DD, the input current of the optical transmitter varies according to a modulation criterion. Recall that, in addition to data transmission, it is necessary to guarantee a constant illumination avoiding flickering [59]. In this sense, the resulting illumination is given by the average of the transmitted signal, i.e., the average output optical power denoted by $P_{\mathrm{avg}}$.

Data transmission for VLC can be categorized as single and multi carrier schemes. Moreover, several modulation schemes can be applied to each of these categories. In the following, a brief description of the modulation schemes available in the state of the art is presented.

##### Single-Carrier Modulations

Single-carrier modulations encode the information in either the position, width or amplitude of the pulses. The modulation scheme referred to as on-off keying (OOK) is one of the simplest and most used, which makes switch input current, and therefore the optical power of the LED, between high and low levels much faster than the human eye can detect [16]. Although it is a very simple modulation, yet is energy- and spectrum-inefficient, it is usually implemented in low cost and medium data-rate applications. There are other simple single-carrier schemes, such as pulse width modulation (PWM), where the information is encoded in the duration of the pulse [60]. Managing the position of the pulse, multi-level pulse position modulation (M-PPM) is proposed in [61]. On the other hand, assuming a constant duration of the pulse while encoding the information in the amplitude of the pulse leads to multi-level pulse amplitude modulation (M-PAM) [62]. In Figure 5, an example of the 4-PAM, OOK, PWM and 4-PPM transmission schemes is depicted considering the dynamic range of the optical transmitter. Each of these single-carrier modulation schemes is subject to a trade-off among distortion, flickering effects, linear dynamic range of the LEDs, transmission bandwidth or bit error rate (BER). In [63], the application of single-carrier modulation schemes in VLC systems is analyzed considering these issues.

##### Multi-Carrier Modulations

Optical orthogonal frequency division multiplexing (O-OFDM) schemes are the most common multi-carrier modulations for VLC, which are variations of the traditional OFDM that is being widely used by current communication systems, such as 5G, 4G, WiFi and digital television [64,65]. However, due to the constraint of generating a real and non-negative transmitted signal for VLC, traditional OFDM schemes for RF transmission cannot be straightforwardly applied. The two main O-OFDM schemes are DC-biased optical–orthogonal frequency division multiplexing (DCO-OFDM) [66] and asymmetrically clipping optical–orthogonal frequency division multiplexing (ACO-OFDM) [67]. Besides, alternative O-OFDM schemes can be found in [68].

Since OFDM schemes are directly based on the fast Fourier transform (FFT) and its inverse (IFFT), generating a real-valued signal requires one to employ some of its properties. In this sense, the input to the IFFT on the transmitter side, i.e., the frequency-domain data, is well-known to be organized in two halves. Specifically, the second half of the frequency-domain data must be the symmetric Hermitian version of the first half. Besides, sub-carriers 0 and N/2 must be 0, where *N* is the number of sub-carriers. This way, due to the Fourier transform properties, the output of the IFFT is real-valued. At this point, there exists a clear difference between DCO-OFDM and ACO-OFDM. For DCO-OFDM, the frequency-domain data, which is denoted as $X_{\mathrm{DCO}}$, can be mathematically expressed as

(1) $$X_{\mathrm{DCO}}=\left[0\;X_{d}\left[1\right]\;X_{d}\left[2\right]\cdots X_{d}\left[N/2-1\right]\;0\;X_{d}{\left[N/2-1\right]}^{*}\cdots X_{d}{\left[2\right]}^{*}\;X_{d}{\left[1\right]}^{*}\right],$$

while for ACO-OFDM, it is given by

(2) $$X_{\mathrm{ACO}}=\left[0\;X_{d}\left[1\right]\;0\;X_{d}\left[3\right]\cdots 0\;X_{d}\left[N/2-1\right]\;0\;X_{d}{\left[N/2-1\right]}^{*}\cdots 0\;X_{d}{\left[3\right]}^{*}\;0\;X_{d}{\left[1\right]}^{*}\right],$$

where ${\left(\cdot \right)}^{*}$ denotes the complex conjugate, and $X_{d}\left[n\right]$ is the frequency-domain data at *n*-th sub-carrier, usually selected from a quadrature amplitude modulation (QAM) constellation point. Notice that only half of the bandwidth can convey useful data. Furthermore, it is worth noting that QAM constellations cannot be applied to single-carrier modulations, since they are subject to consider complex values of the transmitted signal. On the other hand, O-OFDM schemes allow us to use complex-valued constellations in VLC. Then, the IFFT is applied to frequency-domain signal X, where $X_{\mathrm{DCO}}$ and $X_{\mathrm{ACO}}$ are now generalized as X for the sake of simplicity, to obtain the time-domain transmitted signal

(3) $$x_{\mathrm{raw}}=\mathrm{IFFT}\left\{X\right\}.$$

After that, the cyclic prefix is added to obtain

(4) $$x=\left[x_{\mathrm{raw}}\left(N-L_{\mathrm{CP}}:N-1\right)\;x_{\mathrm{raw}}\right],$$

where $L_{\mathrm{CP}}$ is the length of the cyclic prefix.

Once it is ensured that the output of the IFFT is real-valued, the transmitted signal must fit in the dynamic range of the optical transmitter. That is, the transmitted signal ought to be given by non-negative and non-above $P_{\max}$ values (see Figure 5). Beyond this range, the signal is affected by clipping [69].

**Figure 6:**
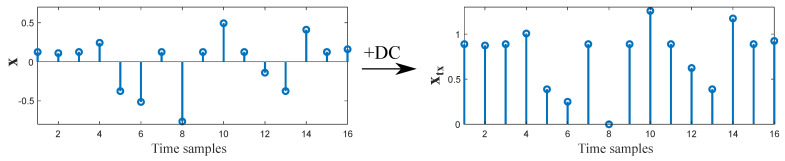
Time-domain DCO-OFDM signal.

For DCO-OFDM, an offset is directly added to the resulting signal after IFFT plus cyclic prefix (see Figure 6), resulting

(5) $$x_{\mathrm{tx}}=x+\mathrm{DC}.$$

As the DC component does not carry any data, DCO-OFDM may be considered as energy-inefficient. However, this is usually not a problem for VLC systems, because a DC value is indeed required for providing a satisfactory illumination level.

**Figure 7:**
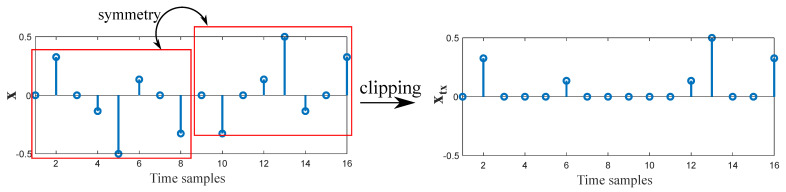
Time-domain ACO-OFDM signal.

Differently, ACO-OFDM makes use of another property of Fourier transform in order to obtain a non-negative signal. By only allocating information in half of the sub-carriers as represented in (2), concretely in the odd sub-carriers, the resulting signal after IFFT can be zero-clipped to eliminate negative values without affecting the information. This distortion only affects even sub-carriers, which had been set to zero. Thus, the transmitted ACO-OFDM signal can be represented as

(6) $$x_{\mathrm{tx}}=\mathrm{clip}\left\{x\right\}\;,$$

where clip denotes the clipping operator that sets to zero the values below zero. The resulting time-domain ACO-OFDM signal is illustrated in Figure 7. Note that the useful number of sub-carriers is N/4, and therefore, the efficiency in terms of bandwidth is reduced by half compared to DCO-OFDM, but the DC offset is not needed. Depending on which system parameters want to be prioritized, either DCO-OFDM or ACO-OFDM should be applied. In this sense, DCO-OFDM provides better spectral efficiency, while ACO-OFDM is more energy efficient [70].

#### 2.1.2. Receiver

In practice, several devices can be used for receiving the transmitted optical power, such as LEDs [23], solar cells [21] or camera sensors [22]. However, for high data rate transmission schemes, the most suitable receiver is the PD because of its large bandwidth, optical responsivity and efficiency in the conversion from light to current [20]. These devices transform the received photons into an electrical current that can be measured and processed by an electronic circuit. Focusing on data transmission, there are three main parameters to consider: responsivity, bandwidth and noise [71,72]. The responsivity is the conversion ratio between the received optical power and generated electric current, and it is expressed as A/W. In contrast to the bandwidth and noise parameters, the responsivity does not depend on the area of detection of the PD. The bandwidth is inversely proportional to the junction capacitance of the PD, which is greater as the area of detection increases and it is given by

(7) $$\Delta f_{PD}=\frac{1}{2\pi R_{\mathrm{load}}C_{j}},$$

where $C_{j}$ and $R_{\mathrm{load}}$ denote the junction capacitance and the load resistance, respectively. Moreover, the resulting noise is proportional to both the area of detection $A_{\mathrm{PD}}$ and the measurement bandwidth $\Delta f_{m}$. Specifically, the noise equivalent power characterizes the sensitivity of the PD, as it is the signal power provided by the PD when signal to noise ratio (SNR) and $\Delta f_{m}$ are unified. It is defined as

(8) $$\mathrm{NEP}=\frac{E_{i}A_{\mathrm{PD}}}{\mathrm{SNR}\sqrt{\Delta f_{m}}}\;\left[\frac{W}{\sqrt{\mathrm{Hz}}}\right],$$

where $E_{i}$ is the incident irradiance expressed in $\frac{W}{{\mathrm{cm}}^{2}}$. Typically, the resulting noise in VLC is modeled as the sum of both shot noise, which depends on the received optical power and the ambient light (mainly daylight), and thermal noise [18].

Optical filters and concentrators are applied to improve the incidence of the optical power into the PD. For thin-film planar filters, the optical response given by a generic angle of incidence φ is [73]

(9) $$T\left(\varphi ;\Delta \lambda ,\hat{\varphi }\right)=\frac{T_{0}}{1+{\left[\frac{\lambda_{0}-\lambda \left(\varphi ;\hat{\varphi }\right)}{\frac{\Delta \lambda }{2}}\right]}^{2\nu }},$$

where $T_{0}$ corresponds to the peak transmission, $\lambda_{0}$ is the wavelength of the transmitted signal, $\frac{\Delta \lambda }{2}$ is the spectral half-power bandwidth, ν is the Lambertian radiation index and $\lambda (\varphi ;\hat{\varphi })$ represents the shifting to shorter wavelengths at non-normal incidences, which is given by $\lambda \left(\varphi ,\hat{\varphi }\right)=\lambda_{0}\left(\frac{n_{s}^{2}-n_{i}^{2}\sin^{2}\varphi }{n_{s}^{2}-n_{i}^{2}\sin^{2}\hat{\varphi }}\right)$, where $n_{s}$ and $n_{i}$ are the effective index of the spacer layer and input layer, respectively, and $\hat{\varphi }$ is the filter orientation. The use of filters following spherical patterns allows us to create optical concentrators that generate optical gain subject to a specific field of view (FoV) [73,74]. The most common are the hemispherical concentrators, i.e., spherical filters truncated at $90^{\circ }$. The gain of a hemispherical concentrator is

(10) $$G\left(\Psi_{c}\right)=\left\{\begin{array}{cc} \frac{n_{s}^{2}}{\sin^{2}\Psi_{c}} & \mathrm{if}\;0\leq \varphi \leq \Psi_{c}\\ 0 & \mathrm{if}\;\varphi >\Psi_{c}, \end{array}\right.$$

where $\Psi_{c}$ denotes the resulting FoV of the PD. Notice that the channel gain increases for narrow FoVs and vice versa. From a networking point of view, wide FoVs increase the reception coverage of each user but also the received interference. This parameter should be carefully handled in the design of the system.

**Figure 8:**
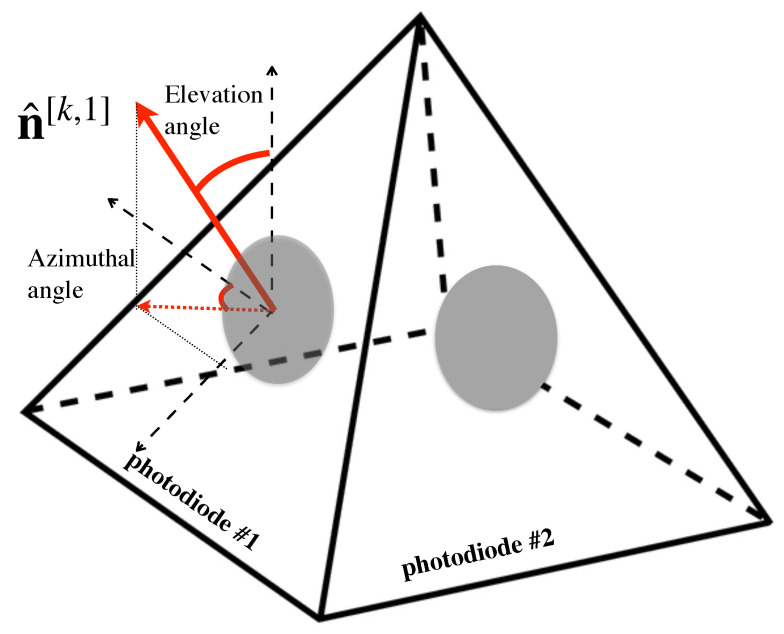
Pyramidal arrangement for an ADR comprising 4 PDs.

**Figure 9:**
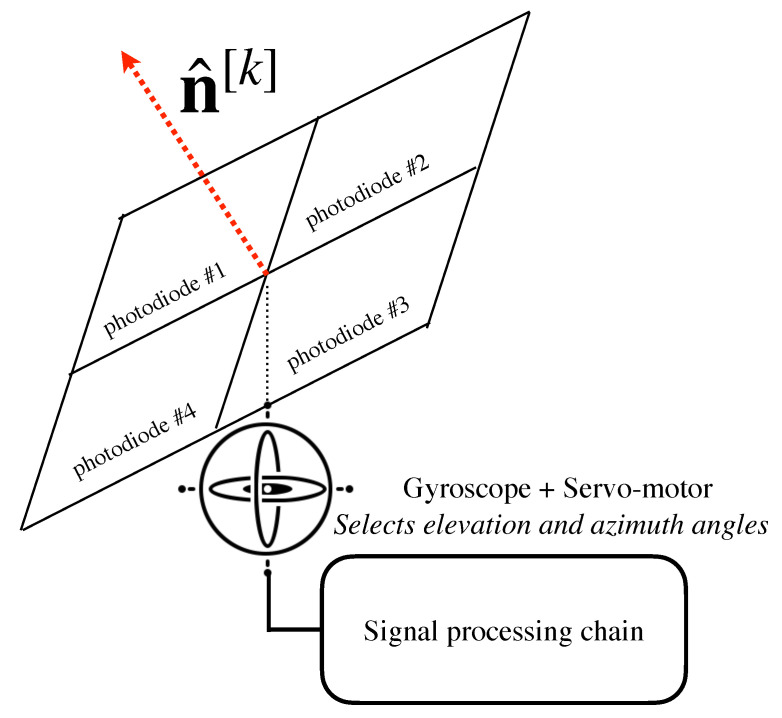
Variable receiving orientation angle photodetector.

Typically, the works in the state of the art consider receivers equipped with a single PD. As described above, there is a trade-off between FoV and optical gain. In this sense, with the aim of providing both wide FoV and optical gain simultaneously, angle diversity receivers (ADRs) have been proposed in several works such as [61,75]. ADRs are composed of several PDs following a geometrical pattern, so that each PD is categorized by an azimuthal and elevation angle, i.e., each PD *p* of user *k* provides an orientation vector denoted by ${\hat{n}}^{\left[k,p\right]}$. Therefore, the PDs of an ADR provide distinct incidence angles for the same optical AP. In [76], the pyramidal and hemispherical arrangements of PDs are analyzed for MIMO channels. For illustrative purposes, Figure 8 shows an ADR following a four faces pyramidal arrangement, where only PDs 1 and 2 are visible, while PDs 3 and 4 are hidden. This way, allocating several PDs around the six faces of a hand-held device and considering the natural movements of the hand, i.e., roll, pitch and yaw, an alternative ADR is proposed in [77]. A variable receiving orientation angle (vROA) photodetector composed of a gyroscope and two servo-motors that modify the azimuthal and elevation angles is proposed in [78] to optimize the orientation of each user, which is denoted by ${\hat{n}}^{\left[k\right]}$ for user *k*. The architecture of the vROA photodetector is depicted in Figure 9. As demonstrated in [76], the use of ADRs improves the performance in terms of sum-rate and BER in MIMO systems. In this sense, the concepts of ADR and vROA photodetector provide a new degree of freedom in the design and the signal processing algorithms [29,79,80,81]. Notice that ADRs improve the visibility of the users in VLC systems and, combined with novel transmission schemes, may be an effective approach for overcoming the LoS-link blocking effects, even without much signal processing.

#### 2.1.3. Backhaul Link

When a multi-cell scenario aims to provide coverage in a large area, there must be a connection (link) among all or part of the APs, which is denoted usually as a backhaul link. A backhaul link is essential in cellular networks [82] and it must be considered and studied also in a realistic way to evaluate the performance of the whole communication system. The backhaul can be either wired or wireless. Different wired technologies for backhaul in VLC have been demonstrated in the literature, such as power line communication (PLC) [83,84,85], Ethernet [86] and optical fiber [87]. However, when the network becomes larger, the use of wired backhaul links imposes an extra cost and cabling infrastructure that sometimes means a high complexity [88]. Furthermore, wired backhauls present a low flexibility when the topology, i.e., location of APs, must be changed. Thus, alternative wireless backhaul links were presented in the literature.

Practical wireless backhaul links have been studied using RF systems [89] or free-space optical (FSO) communications [90,91]. The implementation of VLC or LiFi systems may be complicated when we must connect multiple light fixtures, provided with communication capabilities, by using a wired backhaul. To this end, the first work that studies the feasibility of a wireless backhaul in LiFi systems corresponds to [92], where each AP needs 6 extra LEDs in addition to several PDs for receiving data from neighbouring APs. Later on, new studies such as [45] propose a practical yet sophisticated wireless backhaul link based on VLC reflections. In any case, the backhaul topology must be taken into careful consideration to avoid the system performance decreasing [87].

### 2.2. System Model

**Figure 10:**
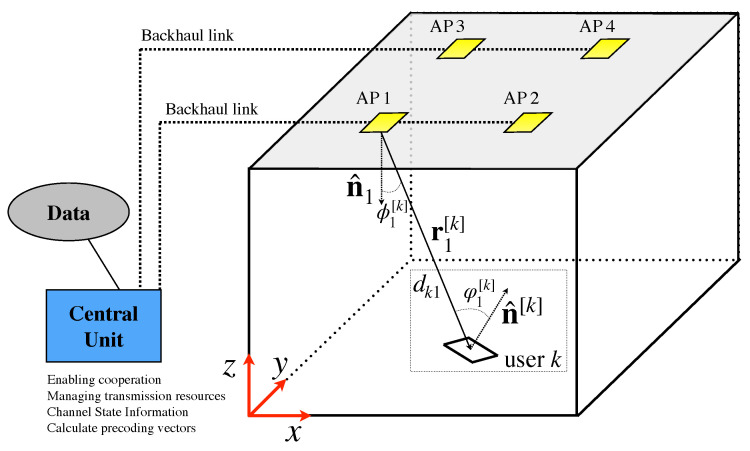
VLC scenario.

Modeling indoor VLC systems considers *M*, m={1,⋯,M}, optical APs providing data service to *K*, k={1,⋯,K}, users, while ensuring constant illumination within the considered indoor aream, as shown in Figure 10. The signal associated with the *M* optical APs at time *t* can be written as $x\left(t\right)={\left[\begin{array}{ccc} x_{1} & \cdots & x_{M} \end{array}\right]}^{T}\in R_{+}^{M\times 1}$, where $x_{m}$ denotes the signal of optical transmitter *m*. Thus, assuming a flat channel response, the signal received by user *k* at time *t* is given by

(11) $$y^{\left[k\right]}\left(t\right)=h^{\left[k\right]}{\left(t\right)}^{T}x\left(t\right)+z^{\left[k\right]}\left(t\right),$$

where $h^{\left[k\right]}\left(t\right)={\left[\begin{array}{ccc} h_{1}^{\left[k\right]} & \cdots & h_{M}^{\left[k\right]} \end{array}\right]}^{T}\in R_{+}^{M\times 1}$ is the channel vector between the *M* optical APs and user *k* at time *t*, $h_{m}^{\left[k\right]}$ corresponds to the channel response between AP *m* and user *k*, and $z^{\left[k\right]}\left(t\right)$ is real-valued additive white Gaussian noise for user *k* with zero mean and variance $\sigma_{z}^{2}$ [18,93].

There are two main components that contribute to the channel impulse response in the time domain of a VLC channel: the front-end and the free-space components. Thus, the time-domain channel impulse response is given by

(12) $$h\left(t\right)=h_{\mathrm{fe}}\left(t\right)⊗h_{\mathrm{fs}}\left(t\right),$$

where $h_{\mathrm{fe}}\left(t\right)$ represents the channel impulse response of the front-end devices that performs a low-pass filtering with a typical 3 dB bandwidth in the range between 3 and 60 MHz [94,95,96]. The frequency-domain channel transfer function of $h_{\mathrm{fe}}\left(t\right)$ can be written as

(13) $$\left|H_{\mathrm{fe}}\left(f\right)\right|=\sqrt{\exp\left(-\frac{f}{F_{\mathrm{fe}}}\right)},$$

where $F_{\mathrm{fe}}$ is the so-called bandwidth factor that controls the frequency-domain characteristics of the front-end devices.

On the other hand, $h_{\mathrm{fs}}\left(t\right)$ denotes the free-space channel impulse response that comprises a flat channel component plus a diffuse channel contribution depending on the propagation conditions. The flat and diffuse components correspond to the LoS and non-LoS (NLoS) propagation, respectively. Moreover, the LoS propagation may be subject to blocking effects [97]. According to [98,99], the channel gain between the optical transmitter *m* and user *k* at time *t* is given by

(14) $$h_{\mathrm{fs},m}^{\left[k\right]}\left(t\right)=\left(h_{\mathrm{LoS},m}^{\left[k\right]}\delta \left(t\right)1_{\mathrm{block},m}^{\left[k\right]}+h_{\mathrm{diff}}\left(t-\Delta T\right)\right),$$

where $h_{\mathrm{LoS},m}^{\left[k\right]}$ denotes the LoS component from optical AP *m* to user *k*, $h_{\mathrm{diff}}$ is the contribution of the NLoS component given by diffuse reflections, ΔT is the delay between the LoS and NLoS components and

(15) $$\begin{array}{c} 1_{\mathrm{block},m}^{\left[k\right]}=\left\{\begin{array}{cc} 1 & \mathrm{if}\;\mathrm{no}\;\mathrm{obstacle}\;\mathrm{between}\;\mathrm{PD}\;k\;\mathrm{and}\;\mathrm{AP}\;m\\ 0 & \mathrm{if}\;\mathrm{obstacle}\;\mathrm{between}\;\mathrm{PD}\;k\;\mathrm{and}\;\mathrm{AP}\;m \end{array}.\right. \end{array}$$

Notice that the LoS component theoretically provides a flat channel response in the frequency domain. However, this flat channel response decays in practice due to the front-end device frequency response ($\left|H_{\mathrm{fe}}\left(f\right)\right|$).

**Figure 11:**
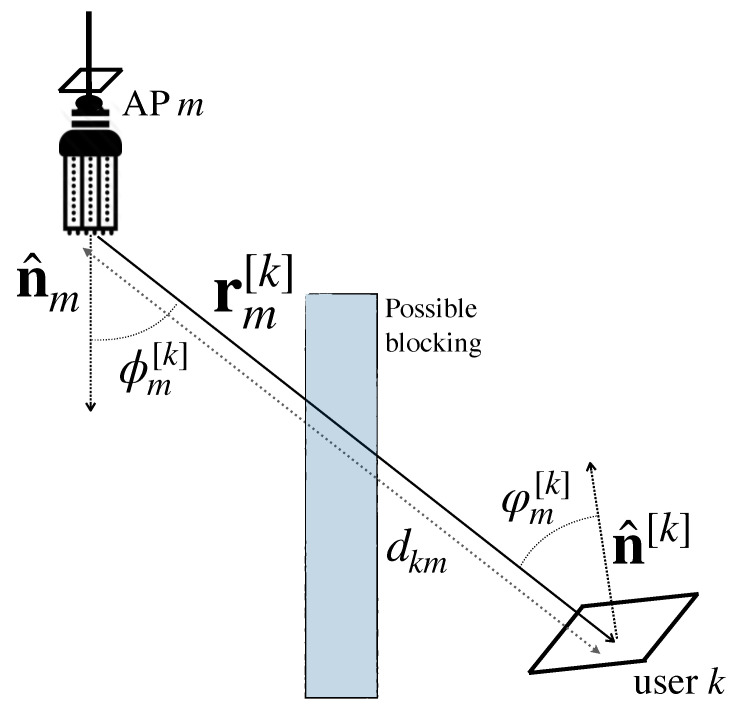
Geometry of the pair transmitter-receiver for the LoS component.

The LoS component is determined by the geometry between the optical AP and the PD, as shown in Figure 11. The distance between optical AP *m* and user *k* is denoted as $d_{km}$ and the irradiance and incidence angles (For ADRs, assuming that the size of the PDs is much smaller than the distance to any optical AP, the irradiance angles between optical AP *m* and all the PDs that compose the ADR of user *k* are the same. On the other hand, distinct incidence angles are obtained in each of the PDs of the ADR of user *k*.) are denoted as $\phi_{m}^{\left[k\right]}$ and $\varphi_{m}^{\left[k\right]}$, respectively. Thus, the LoS contribution to the optical channel between transmitter *m* and user *k* is [61],

(16) $$h_{\mathrm{LoS},m}^{\left[k\right]}=\left\{\begin{array}{cc} \frac{\gamma A_{\mathrm{PD}}}{d_{km}^{2}}R\left(\phi_{m}^{\left[k\right]}\right)T\left(\varphi_{m}^{\left[k\right]}\right)G\left(\Psi_{c}\right)\cos\left(\varphi_{m}^{\left[k\right]}\right) & \varphi_{m}^{\left[k\right]}\leq \Psi_{c}\\ 0 & \varphi_{m}^{\left[k\right]}>\Psi_{c} \end{array}\right.$$

where γ is the responsivity of the PD, $\Psi_{c}$ denotes the FoV, $T\left(\varphi_{m}^{\left[k\right]}\right)$ is the gain of the optical filter (see (9)), $G\left(\Psi_{c}\right)$ is the gain of the hemispherical concentrator (see (10)) and

(17) $$R\left(\phi_{m}^{\left[k\right]}\right)=\frac{\nu +1}{2\pi }\cos^{\nu }\left(\phi_{m}^{\left[k\right]}\right),$$

is the Lambertian beam distribution, where $\nu =\frac{-\log(2)}{\log\left(\phi_{1/2}\right)}$ is the radiation index for the radiation semi-angle $\phi_{1/2}$.

Determining the value of the diffuse component is not straightforward. In this sense, there are two approaches to calculate its behaviour; either decomposing the room surface into a number of reflecting elements [100], or ray tracing the path of a single photon to the PD several times until obtaining an accurate statistical model [98,101]. This second methodology comprises a reasonable computational complexity and it is typically applied in most of the recent works, e.g., [99,102,103]. Thus, assuming that each reflection can be modeled as a first-order spherical radiation, the diffuse component is given by [98],

(18) $$\eta_{\mathrm{diff}}=\frac{A_{\mathrm{PD}}}{A_{\mathrm{room}}}\cdot \frac{\rho_{1}}{1-\rho },$$

where $A_{\mathrm{room}}$ is the area of the room surface, $\rho_{1}$ is the reflectivity of the region initially illuminated by the optical transmitters and ρ is the average reflectivity of the walls. Assuming a uniform value of the reflectivity, the condition $\rho_{1}=\rho$ is typically considered. Then, the diffuse component in the frequency domain is

(19) $$H_{\mathrm{diff}}\left(f\right)=\frac{\eta_{\mathrm{diff}}}{1+j\frac{f}{f_{d}}},$$

where $f_{d}$ is the −3 dB cut-off frequency of the diffuse optical channel.

### 2.3. Scenarios

This survey focuses on indoor scenarios because; (i) LoS-link blockage is more probable in indoors than outdoors; (ii) more complex network topologies are used indoors; (iii) outdoor scenarios present a reduced and spaced number of APs with a higher transmit power, which significantly reduces the cooperation possibilities. At this point, we can clearly identify two main scenarios for indoor VLC systems: single footprint and multiple footprints.

**Figure 12:**
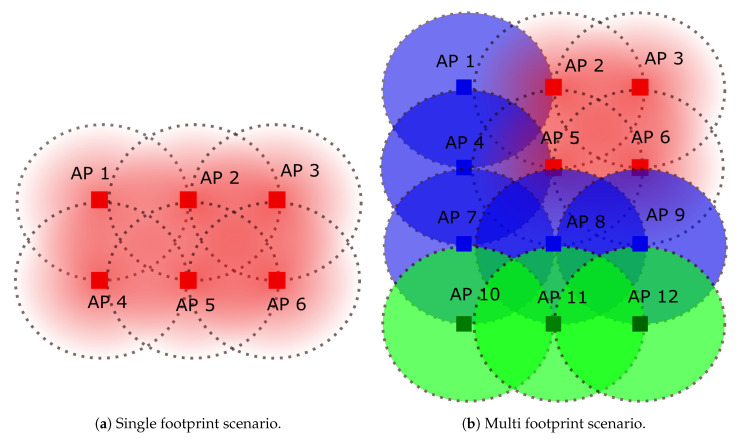
Types of footprint scenarios. Dashed circumferences represent coverage areas rather than illumination areas.

In the first one, the served area, which may correspond to a room, a corridor, etc. is only served by the same resources, either frequency or time. Therefore, the resulting coverage footprint generated by the set of APs is as shown in Figure 12a. This approach is proposed in serveral works of the literature such as [46,104]. In this scenario, the network provides coverage to the whole area with the same set of resources and it seems that the users are served by the same distributed AP. In these scenarios, the interference must be properly managed or handled, yet it is a good opportunity for cooperative schemes. APs may also cooperate to provide a seamless coverage. As will be shown later on, this scenario is usually adopted in coordinated multi-point transmission and reception (CoMP) and some precoding schemes.

Differently, for larger areas, a multiple footprint approach as depicted in Figure 12b is usually considered. In this case, the coverage provided by the set of APs is divided into a different set of resources, either frequency or time. That is, several distributed APs form a coverage footprint, which can be interpreted as coordinated cell or cluster from a cellular perspective.

In these scenarios, cooperation and interference management must be used for each set of APs using same resources, while a seamless transmission should be guaranteed between different footprints. That is, although there are several distributed APs, each of them employing a different set of resources, handover, cooperation and interference management among them and within them must be carried out. Furthermore, these areas can be dynamically created and reconfigured depending on the location of the users, the load of the network, etc. Thus, as pointed out previously, the backhaul is a key element for the management of a multi footprint scenario. It is worth remarking that this scenario is used in some pre-coding schemes, non-orthogonal multiple access (NOMA) proposals [48,105] and user-centric approaches [35,42,106].

## 3. Cooperative Schemes

As previously mentioned, multi-cell VLC systems, i.e., composed of multiple optical APs, are being deployed to create a seamless and reliable network. However, when multiple VLC APs are deployed, the intrinsic overlapping of light coverage areas produces a harmful inter-cell interference as long as cooperative or precoding schemes are not used. On the other hand, this overlapping is required for guaranteeing a proper illumination level satisfying lighting standards.

### 3.1. Introduction to Cooperative Schemes

This section presents a thorough survey of the cooperative VLC schemes, with the main focus on mitigating inter-cell interference as well as overcoming the damaging effect of LoS-link blockage [26]. These two phenomena have been already studied in RF systems, but they present notable differences when working in VLC, and then, they deserve a completely different study.

*Inter-cell interference:* RF networks are typically deployed by qualified staff, minimizing overlapping areas and so decreasing the inter-cell interference. Besides, directional antennas make transmission be under control pointing to a specific area. On the contrary, for VLC, the deployment of the optical APs are usually given and the system aims for providing the lighting infrastructure with communication capabilities. Additionally, VLC networks must comply with lighting standards, which impose the overlap of illumination areas.

*LoS-link blockage:* RF systems are more resilient to blockages due to the intrinsic physical characteristics of such frequency bands, i.e., transmission, reflection, diffraction and refraction, among others. Proper signal power levels can be received even in case of LoS-link blockages between the transmitter and receiver. In contrast, visible light wavelengths cannot go through opaque objects, and the contribution of NLoS links is minimal with respect to LoS.

In the following, we introduce the cooperative schemes presented in the literature to overcome both inter-cell interference and, in some cases, LoS-link blockages.

### 3.2. Cooperative Schemes for VLC

#### 3.2.1. Frequency Reuse (FR)

**Figure 13:**
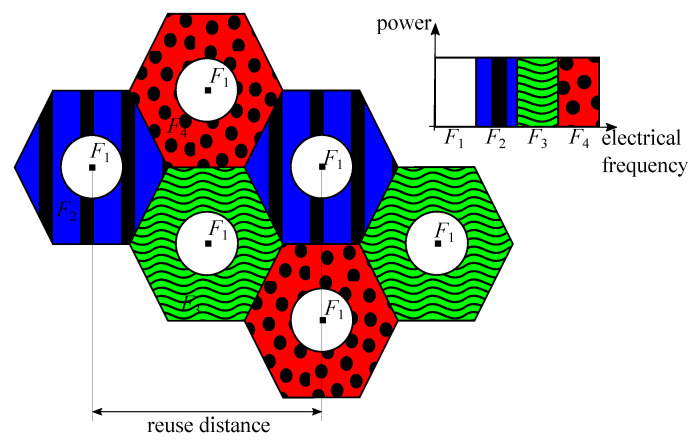
Example of FFR.

FR techniques are straightforwardly applied to VLC systems by simply allocating different frequency sub-bands to neighbouring APs. Note that FR is not strictly a cooperative transmission scheme for providing communication to the same user from multiple APs, but it is a technique that significantly improves the overall system performance by allocating different resources among multiple APs, and therefore, reducing the inter-cell interference. For the sake of completeness, FR techniques must be considered in this survey. Two optical APs that transmit at the same frequency band are separated by the reuse distance. As users located at the cell-edge areas are the ones subject to strong inter-cell interference, more efficient FR techniques were proposed traditionally in RF multi-cell systems, such as fractional frequency reuse (FFR), which employs different frequency reuse factors in cell-center and cell-edge areas to keep the inter-cell interference under control [107,108]. FFR is evaluated in VLC scenarios by the authors of [109,110], demonstrating that the inter-cell interference can be reduced notably for those users located at the cell-edge areas. An example of FFR network in a VLC scenario is shown in Figure 13. Note that users located at the cell center are all served by the same frequency band, assuming that the inter-cell interference among them is low. Then, cell-center area has a frequency-reuse factor of 1. However, users located at the borders of the cell are served by different frequency bands, with a frequency reuse factor of 3 in the example.

A potential limitation of this technique is notable in ultra-dense VLC networks, where users located at the cell-center areas also suffer from a high inter-cell interference level coming from adjacent lamps [111]. That is, managing the transmission resources, e.g., allocating frequency sub-bands in an O-OFDM scheme, is more complex and less efficient as the network becomes more dense.

#### 3.2.2. Joint Transmission—Coordinated Multipoint (Jt-Comp)

CoMP transmission schemes were firstly proposed to multi-cell RF communication systems subject to strong inter-cell interference. The main difference with respect to FR techniques is that there are no divisions in the frequency bands for allocating them to APs, and then, the area is not divided in advance for serving users over specific frequency bands. The fundamentals of JT-CoMP is the coordination of multiple APs in order to serve users in the same time and frequency resources in a cooperative manner. This way, neighboring APs are no longer interference sources, since using JT-CoMP converts the inter-cell interference coming from different optical APs into useful signals [112,113]. These methods can be grouped into the single footprint scenario.

Transmission techniques based on CoMP are originally proposed in long term evolution(LTE) [114] and LTE advanced [115] for improving the cell-edge throughput in multi-cell scenarios. Early studies employed directional antennas in order to improve the performance of JT-CoMP in RF [116] and VLC [117] systems, where, in the latter case, the APs are equipped with non-conventional directional LEDs pointing to the cell-edge areas. Similarly to the approach considered in FFR, CoMP techniques are also proposed to cooperatively serve users only located at cell borders [117]. To provide such a network in VLC, sophisticated APs must be used, where some LEDs are pointing downwards to the center and some other are facing to the cell borders. This may be difficult to deploy, especially guaranteeing homogeneous illumination.

**Figure 14:**
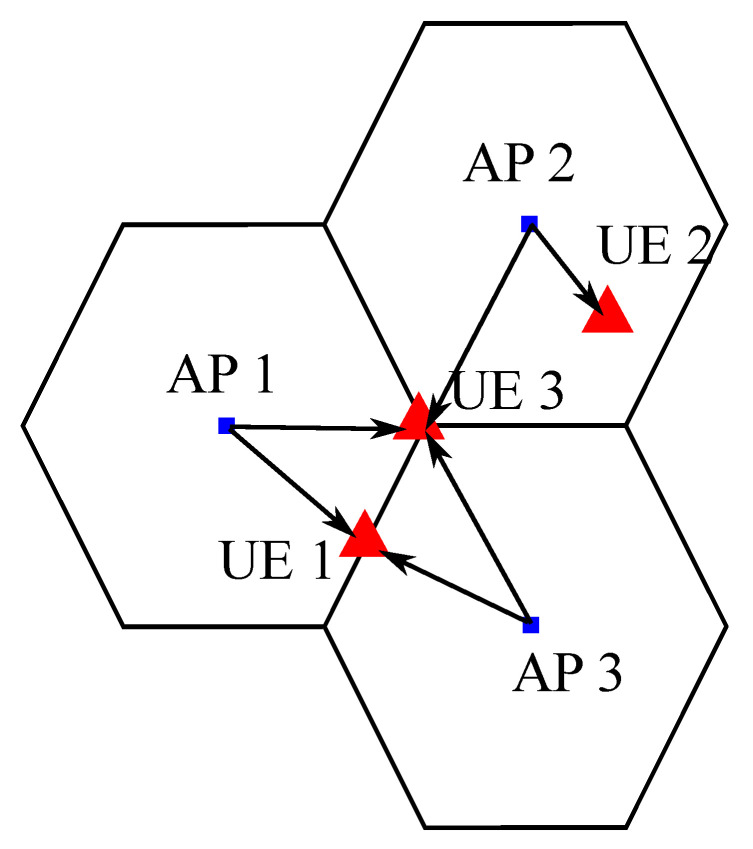
Toy example of JT-CoMP.

In this context, JT-CoMP techniques have gained importance in VLC because, in addition to managing the inter-cell interference, they also mitigate the LoS-link blockage problem that the technology experiences [118,119]. Now, the LoS-link blockage with one AP is not as dramatic as when users are served by one only AP. An illustration of JT-CoMP is given in Figure 14, where multiple APs are serving the same users. Note that the user equipment (UE) 2 is served only by AP 2 as its location allows it to get a great coverage by receiving information from its nearest AP. However, UE 1 and UE 3 are located at cell edge, where cooperation among neighbouring APs is considered in order to improve their performance. In these cases, cooperation among 2 or 3 APs is required, respectively.

In this case, CoMP techniques have also been demonstrated to be helpful for enabling smoother handover operation among neighbouring VLC APs [120].

In [121], the authors present an analytical model of a multi-user VLC scenario where JT-CoMP techniques are employed. It is demonstrated that JT-CoMP also improves the coverage performance of the network. Moreover, in this work, two ways of invoking JT-CoMP techniques are proposed; non-coherent and coherent. In the former, multiple APs transmit the same information to the users without any correction or synchronization, and therefore, the signal power levels received from multiple optical APs are added at the receiver [113,122]. On the contrary, in the coherent technique, the cooperating APs share the CSI with users. Satisfying this condition allows us to implement precoding techniques, which are described in detail in the following section together with synchronization signaling in order to provide multiplexing gain, i.e., transmitting several symbols over the same transmission resource; either time of frequency, canceling or minimizing the interference among them [113,122]. It is demonstrated that JT-CoMP coherent technique provides higher performance gain in comparison with JT-CoMP non-coherent techniques, at the expense of a higher complexity and feedback.

When the VLC system is fully optical, the LoS-link blockage may affect both uplink and downlink channels, without having the option of communicating feedback information from a blocked user for the whole duration of the blockage event. As VLC-enabled devices may not have the chance to feed their channel condition back to the optical APs frequently, particularly when the number of devices to be served per small cell is too large, it is desirable that the resource allocation scheme of the multi-cell VLC systems corresponds to a pre-defined strategy that provides as good coverage as possible.

#### 3.2.3. Pre-Defined Jt-Comp

In general terms, the use of CoMP in VLC has demonstrated a higher level of reliability. However, most of the CoMP techniques proposed in the literature so far for VLC are optimized ad hoc for the set of active users and, due to that, they have a computational complexity that increases with the number of users. Besides, these solutions are not static, making the communication unstable and unreliable when LoS-link blockages with relative short duration happen regularly, e.g., when obstacles pass by quickly. These techniques demand to sense the optical wireless channel permanently and to reallocate resources accordingly if a blockage event is detected; in addition, since the blockage also affects the feedback channel, the users may not have the chance to inform the serving AP for fully optical VLC systems about this situation.

This way, pre-defined JT-CoMP systems are proposed in the literature, whose fundamentals are to keep the same transmission resources over time, and to pre-define the allocation of transmission resources in an efficient way depending on the characteristics of the scenario. Firstly, a simple pre-defined JT-CoMP scheme is proposed in [104] and applied to a corridor scenario. A time division multiple access (TDMA) is employed, where the time domain is divided into three time slots and each of them assigned to a different AP. Each user is served by three different APs, which shows a robust and reliable coverage against LoS-link blocking elements. Later on, the authors of [46] extend the previous work to an O-OFDM system with a hexagonal cell deployment, which comprises large indoor scenarios such as conference rooms or laboratories. Notice that TDMA is no longer used for resource allocation as it decreases the system performance notably. Therefore, frequency sub-bands and I-Q signals may be defined as resources to be allocated.

General guidelines for creating pre-defined JT-CoMP schemes are published in [31], where multi-chip LEDs and O-OFDM are proposed in order to increase the degrees of freedom, i.e., the transmission resources. LED color and frequency sub-bands are the two variables defining the allocation resources. Note that I-Q signals are no longer considered independently in order not to decrease the system performance. The number of resources is defined as G=|C|·|F|, where |C| is the number of chip colors of an LED and |F| is the number of frequency sub-bands per color. The cluster size is Q=G/|S|, where |S| is the number of sectors per cell. The number of optical APs that can cooperate to serve users simultaneously by means of JT-CoMP is also defined, and according to all these concepts, a JT-CoMP network is pre-defined.

**Figure 15:**
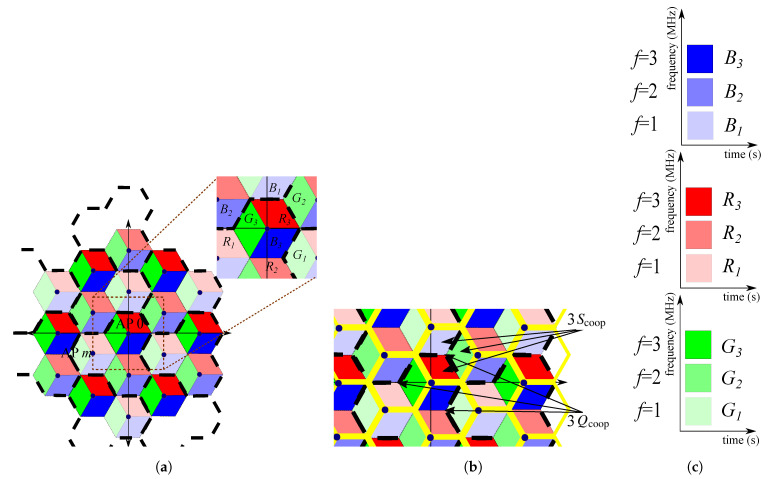
Example of pre-defined JT-CoMP showing a resource allocation for a multi-cell VLC network with RGB LEDs (|C| = 3), three sectors per cell (|S| = 3), and three electrical frequency sub-bands per color (|F| = 3). Cell clusters and cooperative sets are shown. $S_{\mathrm{coop}}$ represents the number of sectors within a cooperation area and $Q_{\mathrm{coop}}$ is the number of cooperation areas where an AP contributes. (**a**) Resource allocation scheme suitable for single-cell transmission. Dashed black line represents cell clusters (Q = 3); (**b**) Cooperation strategy with 3 cooperative APs per cooperating area (solid yellow lines); (**c**) Orthogonal resources.

An example of pre-defined JT-CoMP network is presented in Figure 15, where a two-tier VLC multi-cell network is represented. RGB LEDs are used (|C| = 3), cells are virtually divided into 3 sectors (|S| = 3) and the electrical bandwidth of every color chip is divided into 3 sub-bands (|F| = 3) that are represented with different brightness levels. Thus, depending on the location, the user must decode the coming signal at color and sub-band that correspond to such sector. It is worthy to mention that the authors of [31] provide the guidelines for the creation of pre-defined JT-CoMP guaranteeing a homogeneous signal-to-interference-plus-noise ratio (SINR), i.e., the spatial SINR distribution at every sector of the network is the same, with and without cooperation among neighbouring APs. It is demonstrated that JT-CoMP increases the mean cell data rate with respect to single-AP transmission in both cases, with and without LoS-link blockages.

One of the presented pre-defined JT-CoMP schemes is thoroughly studied in [111] for a scenario where the orientation of the receiver, i.e., the PD, and the density of obstacles vary. This work shows the robustness and reliability of pre-defined JT-CoMP schemes by reducing the outage probability and increasing average cell data rate considerably.

It is worth recalling that all the techniques that were previously presented here need a backhaul approach that is often assumed to be ideal [123], i.e., lossless, noiseless and instantaneous, and sometimes they are supposed to involve unlimited capacity [124], which is an unrealistic scenario. That is, the backhauling should not be underestimated when referring to cooperative transmission techniques.

#### 3.2.4. Relay-Based Cooperative Techniques

In this context, relay-assisted techniques are considered as suitable alternatives to support the main link and improve the system throughput. In [125], a cooperative VLC system is proposed using an intermediate light source that acts as a relay terminal. A relay technique using an asymmetrically clipped direct current biased optical OFDM (ADO-OFDM) is proposed in [126]. This way, with the aim of relaying information and transmitting its own data using odd and even subcarriers, respectively, a relay-assisted VLC system comprising a main light on the ceiling and one task light on a table is presented in [127]. Notice that these relay-based cooperative techniques can be considered as special cases of the single footprint scenario.

**Figure 16:**
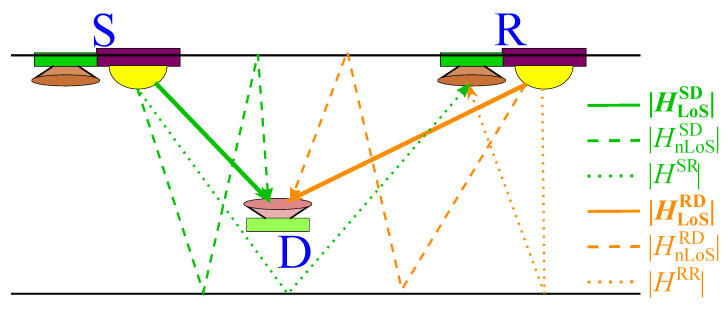
Relay-assisted technique using reflections [45].

The applicability of these schemes to more flexible scenarios, in which even portable sources of light are involved, may not be feasible. A cooperative VLC system based on relay-assisted techniques where reflections are used as relaying links is proposed in [45] assuming that a PD is added per each AP. This proposal increases the reliability of the VLC networks in the presence of blocking elements, as it is common in indoor scenarios. That is, it provides an additional link that allows us to maintain the system performance even with the blockage of the LoS link. The basics for reflection-based cooperative techniques are presented in [45]. These techniques comprise a source S, a relay R and a destination user D, as depicted in Figure 16. Note that $H^{\mathrm{SR}}$ is the reflection-based channel where neighbouring APs are communicated. Then, *R* can relay the information to *D* after adequate processing based on amplify-and-forward and decode-and-forward methods and half-duplex and full-duplex modes.

#### 3.2.5. Other Techniques

NOMA was firstly proposed for VLC as a proper technology to boost the narrow modulation rate available for VLC systems [128]. Users are multiplexed in the power domain by using coding on the transmitter side and successive interference cancellation at the receiver. Later on, NOMA is proposed as an adequate technique for providing a fair coverage, regardless of user location, i.e., users located at the cell edge are benefited thanks to NOMA and the received inter-cell interference is reduced [48,105,129]. These NOMA techniques can be managed as single footprint scenarios.

Spatial division multiple access (SDMA) can be considered as a clustering technique to reuse resources at a different group of users. SDMA may reduce the inter-user interference, but its greatest advantage is the reuse of multiple access resources such as time and frequency. Most of the state-of-the-art SDMA techniques for VLC involve the fabrication of a new transceiver capable of transmitting multiple beams, which increases the cost and complexity considerably [130,131]. However, authors of [132] recently proposed a novel SDMA technique for MIMO-VLC systems that do not need a new transceiver design, and they demonstrate the superiority of SDMA against traditional OFDMA. On the contrary, SDMA techniques are classified into the multi footprint category.

Both NOMA and SDMA can be combined for improving the performance of cell-edge users in a multi-cell VLC network [133]. Besides, SDMA may be employed as a grouping technique prior to JT-CoMP techniques [134].

To conclude this section, Table 3 represents a summary of the previously described cooperative techniques in VLC. As can be seen, some of them focus on interference mitigation, whereas some others address the LoS-link blockage by providing additional ones. The choice of the cooperative technique must be determined depending on the scenario, permitted complexity and the target of the system.

## 4. Precoding Schemes

Since several LED lamps are typically deployed in an indoor scenario in order to provide constant and uniform illumination, each user may receive a useful signal from multiple optical APs. Thus, downlink VLC lends itself naturally to MIMO systems. In this sense, a vast amount of works focused on exploiting the benefits of MIMO channels have been derived for RF systems. At this point, the upcoming question may be formulated; Is the well-known MIMO signal processing for RF systems, e.g., [33,135,136], applicable to VLC systems?. The general answer is; yes, but it is not straightforward. In the following, we provide an introduction to precoding schemes, including a review of the issues to be solved for implementing precoding signal processing in VLC, as well as concepts about the capacity of VLC MIMO systems. After that, a review of precoding schemes for VLC derived from the state of the art is detailed.

### 4.1. Introduction to Precoding Schemes

#### 4.1.1. Fundamentals

**Figure 17:**
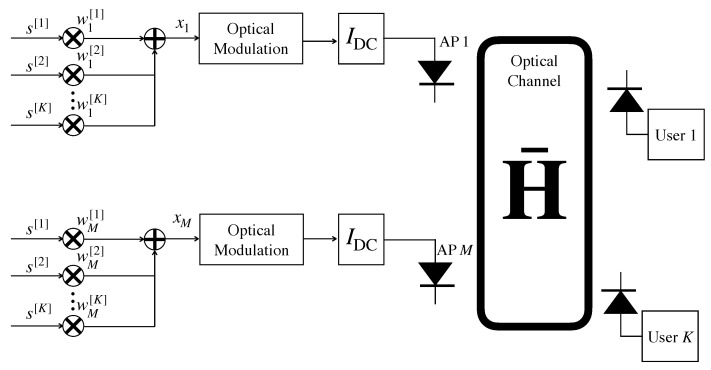
Schematic diagram for precoding schemes in a MU-MIMO VLC system.

Again, let us consider a scenario comprising *M* optical APs and *K* users where K≤M. If K≥M, a scheduling strategy can be implemented, so that in each transmission resource, either time or frequency, the number of served users is less or equal than the number of optical APs. For the sake of generality, transmission over a flat frequency channel (Single carrier transmission occurs in the flat section of the optical bandwidth and multi-carrier transmission provides a flat frequency channel response at each subcarrier.) is assumed from now on, and therefore, the following derivations can be straightforwardly applied to the time and frequency domains. Besides, notice that the concepts described in this introduction are not specific for VLC, but they can be considered for any MIMO system. The symbols intended to the set of users are given by the vector $s={\left[\begin{array}{ccc} s^{\left[1\right]} & \cdots & s^{\left[K\right]} \end{array}\right]}^{T}\in C^{K\times 1}$ where $s^{\left[k\right]}$ is the symbol intended to user *k*. Moreover, the transmitted signal is denoted by $x={\left[\begin{array}{ccc} x_{1} & \cdots & x_{M} \end{array}\right]}^{T}\in C^{M\times 1}$ where $x_{m}$ is the signal of the optical AP *m*. For linear precoding schemes, each optical AP composes a linear combination of the symbols intended to the *K* users, as seen in Figure 17. That is,

(20) $$x_{m}=e_{m}^{T}\left(w^{\left[1\right]}s^{\left[1\right]}+\cdots +w^{\left[k\right]}s^{\left[k\right]}+\cdots +w^{\left[K\right]}s^{\left[K\right]}\right),$$

where $w^{\left[k\right]}={\left[\begin{array}{ccc} w_{1}^{\left[k\right]} & \cdots & w_{M}^{\left[k\right]} \end{array}\right]}^{T}\in C^{M\times 1}$ is the precoding vector associated to user *k* and $e_{m}$ is the unit column vector whose *m*-th entry is 1. Thus, the signal received at user *k* can be written as

(21) $$y^{\left[k\right]}=\underset{\mathrm{desired}\;\mathrm{symbol}}{\underset{︸}{{h^{\left[k\right]}}^{T}w^{\left[k\right]}s^{\left[k\right]}}}+\underset{\mathrm{interference}}{\underset{︸}{{h^{\left[k\right]}}^{T}\sum_{j=1,j\neq k}^{K}w^{\left[j\right]}s^{\left[j\right]}}}+\underset{\mathrm{noise}}{\underset{︸}{z^{\left[k\right]}}},$$

where $h^{\left[k\right]}={\left[\begin{array}{ccc} h_{1}^{\left[k\right]} & \cdots & h_{M}^{\left[k\right]} \end{array}\right]}^{T}\in C^{M\times 1}$ is the channel from the *M* optical APs to user *k*. Denoting the signal received by the *K* users as $y={\left[\begin{array}{ccc} y^{\left[1\right]} & \cdots & y^{\left[K\right]} \end{array}\right]}^{T}\in C^{K\times 1}$, we can express it in a matrix format as

(22) $$\begin{array}{cc} y & =\bar{H}x+z\\ \; & =\bar{H}Ws+z, \end{array}$$

where $\bar{H}={\left[\begin{array}{ccc} h^{\left[1\right]} & \cdots & h^{\left[K\right]} \end{array}\right]}^{T}\in C^{K\times M}$ is the channel matrix, $W=\left[\begin{array}{ccc} w^{\left[1\right]} & \cdots & w^{\left[K\right]} \end{array}\right]\in C^{M\times K}$ is the precoding matrix and $z={\left[\begin{array}{ccc} z^{\left[1\right]} & \cdots & z^{\left[K\right]} \end{array}\right]}^{T}\in C^{K\times 1}$ is the vector containing the noise of the *K* users.

So far, only the basic system model for precoding schemes has been described. Several criteria can be considered for determining the value of the precoding vectors. Zero forcing (ZF) precoding is subject to completely cancelling the multi-user interference (see (21)), i.e., $h^{\left[k\right]}w^{\left[j\right]}=0,\forall j\neq k$, considering specific conditions such as maximizing the sum-rate [33,137], guaranteeing the fairness among users [135] or optimizing a specific objective function [138]. The precoding vectors can also be determined by minimizing the mean squared error (MSE) between the received symbol of each user and the legitimate transmitted symbol [139,140]. In this context, maximum ratio combining (MRC) on the transmitter side can also be considered [141]. However, there are still some issues to solve before considering the implementation of these transmission schemes in VLC.

#### 4.1.2. Requirements for VLC Precoding Schemes

In the following, the requirements for deploying VLC precoding schemes are presented:

*Uplink and CSI:* VLC systems naturally consider a frequency division duplex (FDD) operation mode. Although downlink typically occurs in the visible optical domain, infrared (IR) communications [142,143] or umbrella RF networks such as WiFi or femtocells [144,145] are considered for implementing the uplink transmission. Notice that time division duplex (TDD) requires one to transmit both downlink and uplink through the visible optical domain, which may be annoying for practical implementations. Thus, the first issue that must be solved for implementing precoding techniques is to provide CSI in order to determine the precoding matrix W. In [146,147], the cost of providing CSI in MIMO networks is analyzed. First, *P* orthogonal estimation pilots are transmitted comprising *P* dedicated time/frequency slots. The downlink channel estimated by each of the *K* users is then fed back through the uplink to the optical APs. To conclude, once the precoding vectors are calculated, pilots considering the precoding vectors are transmitted in an orthogonal fashion for coherence detection. Let us denote the fraction of downlink transmission resources, either time or frequency, allocated for estimation pilots and coherence detection as $\theta_{\mathrm{ep}}$ and $\theta_{\mathrm{cd}}$, respectively. Similarly, the fraction of uplink transmission resources for channel feedback is denoted as $\theta_{\mathrm{fb}}$. Therefore, the efficiency of the user-rate for precoding schemes is penalized by a factor $\eta_{p}=1-\left(P\left(\theta_{\mathrm{ep}}+\theta_{\mathrm{cd}}\right)+K\theta_{\mathrm{fb}}\right)$. It is worth remarking that this penalty must be taken into consideration when comparing precoding schemes with blind transmission schemes such as orthogonal resource allocation [109] or blind interference alignment [29].

*Backhaul:* Data sharing among optical APs is required for implementing precoding schemes. As can be seen in (20), each optical AP knows the symbols intended to the *K* users and generate a transmitted signal given by a linear combination of the precoding vectors and these symbols. Notice that this fact makes the network system based on precoding schemes more resilient to the blockage of one or several links between any pair optical AP user, as discussed below. As described in the previous section, multiple approaches can be considered for providing backhauling in VLC, such as PLC [83,84], Ethernet [86], optical fiber [87] and wireless, either optical or RF communications [45,90,91,92]. Thus, precoding schemes require one to implement a central unit (CU) to generate the symbols intended to the users, manage the pilot transmission, obtain the CSI knowledge and calculate the precoding vectors, as can be seen in Figure 10.

*Transmission within the LED dynamic range:* As described in Section 2, the transmitted signal in the optical domain for IM/DD must correspond to a real and non-negative value. However, canceling or simply managing the interference by using linear precoding implicitly generates negative values. Hence, once the precoded signal is generated, a DC bias current is added at each optical AP to ensure the non-negativity of the signal. However, this condition may lead to clipping noise when the resulting signal does not fit in the dynamic range of the LED transmitters (see Figure 5). The scheme for implementing a precoding scheme in VLC is shown in Figure 17.

#### 4.1.3. Capacity of Mimo VLC Systems

The capacity of the additive white Gaussian noise (AWGN) channel was derived by Shannon in [36,37]. For MIMO systems based on RF transmission, the capacity has been studied in several works [148,149,150]. In this sense, non-linear transmission schemes such as dirty paper coding (DPC) are capacity achieving, although subject to a high computational complexity [151]. In this context, a common oversight is to consider the Shannon capacity straightforwardly for analyzing the performance of precoding schemes in VLC.

In [40], the capacity of the single-input single-output (SISO) free-space optical intensity channel is upper and lower bounded. Similarly, the upper and lower bounds of the optical channel considering IM/DD with PAM are derived in [38]. This work is extended in [39], considering non-uniform input distribution for PAM transmission. These works provide tight bounds for a single optical link at low and high SNRs. Assuming parallel optical channels, i.e., non-interfering channels between the same pair transmitter-receiver employing multiplexing techniques such as TDMA, the resulting lower and upper bounds of the capacity are derived in [152]. For precoding schemes applied to VLC, a lower bound of the capacity is derived in [153]. Specifically, the lower bound of the capacity for user *k* is

(23) $$C^{\left[k\right]}\geq \frac{1}{2}\log\left(1+\frac{2|{h^{\left[k\right]}}^{T}w^{\left[k\right]}{|}^{2}}{\pi e\left(\frac{1}{3}\sum_{j\neq k}{\left|{h^{\left[k\right]}}^{T}w^{\left[j\right]}\right|}^{2}+\sigma_{n}^{2}\right)}\right).$$

Considering exclusively ZF precoding, i.e., ${h^{\left[k\right]}}^{T}w^{\left[j\right]}=0,\forall k\neq j$, the specific lower bounds and an alternative methodology for obtaining the ZF precoding vectors based on solving a specific optimization problem are derived in [34].

**Figure 18:**
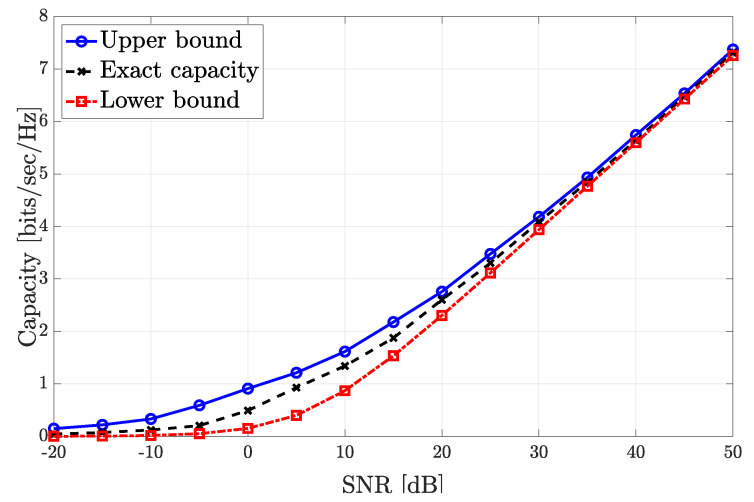
Exact, upper and lower capacity bounds for amplitude constrained Gaussian channels.

For illustrative purposes, the exact, lower and upper bounds of the capacity assuming ZF precoding versus the peak SNR of an amplitude constrained Gaussian channels are shown in Figure 18. It can be seen that the lower bound of the capacity provides a good approximation of the exact capacity. Moreover, mathematically, the lower bound results in being easier to manage than the upper bound derived in [40,153], which requires one to solve an optimization problem for satisfying the amplitude constraint.

### 4.2. Precoding Schemes for VLC

To the best of our knowledge, the first work that considers precoding for optical wireless communications is [154]. In this sense, the achievable data rates for MIMO optical wireless communications are analyzed in [32,155]. These works provide the fundamentals of the implementation of MIMO techniques in the optical domain. In [155], it is shown that assuming a 5 m × 5 m × 3 m room, the maximum delay between LoS components corresponds to 10 nsec. That is, the non-LoS component can be neglected for a transmission bandwidth up to 100 MHz for this scenario, and therefore, the small-scale effects do not generate uncorrelated channel responses, as usually occurs in RF systems. However, the performance of a MIMO optical link is penalized by the correlation among the channel responses. This issue is one of the main drawbacks for implementing precoding techniques in VLC systems. It is worthy noticing that the use of precoding techniques is not considered in these works, i.e., [32,155]. Furthermore, in this survey, we focus on linear precoding schemes and their comparison with DPC [151,156]. Recall that DPC achieves the capacity by completely canceling the interference in a non-causal manner, which is subject to a huge computational complexity, so that its implementation cannot be considered in a realistic VLC system.

Each optical AP generates a linear combination of the symbols intended to the *K* users considered in the implementation of a precoding scheme. This condition can be easily checked in (20). As a consequence, the symbols intended to user *k* are received for the optical APs 1,⋯,l−1,l+1,⋯L, even if the channel between user *k* and optical AP *l* is blocked. Therefore, the use of precoding schemes may be considered as an effective technique for overcoming the shadowing and blocking effects. In this sense, for precoding schemes, the transmission of *K* independent symbols simultaneously is subject to obtain a full rank channel matrix (see (22)), i.e., $\mathrm{rank}(\bar{H})=K$. It is worth recalling that increasing the number of optical APs for a fixed number of users may contribute to satisfy this condition.

The performance of precoding schemes for overcoming the blocking and shadowing effects is analyzed in [157]. In such a way, the authors propose an alternative precoding scheme for minimizing the effects of the blocked channels. The use of ADRs combined with max-min SINR precoding is proposed in [158]. It is shown that ADRs are useful for avoiding the blocking effects. Furthermore, blocking effects have a direct impact on the estimation of CSI, which is required for implementing precoding schemes. Robust precoding schemes considering partial, outdated or stale CSI are proposed in [140,157,158,159]. However, the implementation of precoding schemes in VLC systems subject to blocking and shadowing effects is still and open issue, as described in Section 6. In the following, we describe the implementation of distinct precoding schemes for VLC.

#### 4.2.1. Linear ZF Precoding

First, it is worth recalling that linear ZF precoding completely cancels the interference coming from transmission to all other users, i.e., assuming a received signal according to (21), ZF precoding involves ${h^{\left[k\right]}}^{T}w^{\left[j\right]}=0,\forall k\neq j$. Therefore, the resulting system corresponds to *K* parallel non-interfering channels, each with a specific channel gain. In [160], both linear ZF precoding and DPC are considered for VLC assuming the Shannon capacity equation. Interestingly, it is shown that ZF linear precoding obtains a performance close to the optimal one given by DPC. However, this work does not consider the correlation among the channel responses of the users. Assuming users equipped with multiple PDs characterized by distinct FoV each, the implementation of block diagonalization (BD) in a VLC system is proposed in [161]. For BD, each user cancels the interference coming from transmission to other users in blocks with a size equal to the number of PDs allocated to each user. After that, the interference among the PDs of the same user can be cancelled considering a postcoding matrix. This approach is also considered for a two-user scenario in [162].

Assuming MRC at the multiple PDs of each user, the precoding vectors are calculated subject to obtaining a minimum rate for both users. Determining the precoding vectors for ZF typically requires inversion of the channel matrix. In [34], the performance of ZF precoding is analyzed considering the upper and lower bound of the capacity for VLC and the constraints given by the optimal channel, i.e., a real and non-negative transmitted signal. After that, a methodology for calculating the precoding vectors through convex optimization instead of inverting the channel matrix is derived. Furthermore, this work also considers both criteria maximizing the sum-rate and max-min fairness subject to ZF precoding.

#### 4.2.2. Minimum Mean Squared Error (Mmse)

Although ZF precoding completely cancels the interference, it may provide a low performance, especially at low SNR, since the resulting channel gains do not consider criteria such as fairness of BER. At this point, the MMSE precoding minimizes the error between the received and legitimate transmitted signal, which results in minimizing the BER. Assuming CoMP among optical transmitters, MMSE precoding in a MU-MISO VLC system subject to the constraints given by the optical channel is proposed in [118]. Moreover, this work considers both perfect and uncertain CSI given by the mobility of the users. In [163], MMSE precoding is derived considering the optical and power constraints for maintaining stable brightness. Instead of using the traditional pseudo-inverse methodology, the precoding vectors are determined by solving a convex optimization problem [164]. It is shown that MMSE precoding outperforms ZF precoding when considering the optical constraints. Focusing on a single-user MIMO optical link, MMSE precoding applying singular value decomposition at the transmitters and maximum likelihood detection on the receiver side is analyzed in [165]. After that, an iterative method for calculating the MMSE precoder is derived. This iterative approach is a practical solution assuming a static scenario, since the complexity of this algorithm requires one to converge to a practical solution. Interestingly, this work shows that the MMSE precoding provides a poor BER when the illumination requirements are close to the lower or upper bound of the dynamic range of the optical transmitters. In this case, these techniques can be classified as multi footprint.

#### 4.2.3. Other Precoding Techniques

Other precoding designs consider criteria such as solving the max-min SINR. In [166], this approach is proposed using optimal linear precoding while calculating the precoding vectors through convex optimization. However, this solution is subject to a high complexity. An iterative methodology is also proposed in [166], in order to relax the complexity of the optimal solution. This way, the max-min SINR criterion is also considered [158]. This work considers ADRs so that each receiver is composed of several PDs allocated according to a geometrical pattern [76].

#### 4.2.4. Non-Perfect CSI

As also occurs in RF systems, one of the main challenges for implementing precoding schemes in VLC systems is to provide accurate and non-stale CSI. Although the small-scale effects are often negligible in VLC systems, the mobility of the users and the presence of blocking objects may generate CSI variability. In [167], the performance of DPC, ZF and BD is analyzed for VLC systems subject to non-perfect CSI. They propose a CSI error model where the estimated channel vector of each user is some degrees out of the real value. That is, the CSI is simply not perfect and other considerations about its accuracy or freshness are not taken into account. More specific uncertainty models such as noisy and outdated CSI are considered in [118] for determining the MMSE precoding. The outdated CSI uncertainty model is also considered for max-min SINR precoding in [158]. In [168], the max-min criterion is applied to the capacity bounds subject to outdated CSI for calculating the precoding vectors. All these works show that precoding schemes are considerably penalized by non-perfect CSI. However, none of them propose to exploit the deterministic behaviour of the optical channel to minimize the impact of the CSI uncertainty, which is identified as an open issue for VLC systems.

#### 4.2.5. Blind Interference Alignment

The lack of CSI at the transmitters usually means the use of orthogonal resource allocation schemes such as TDMA. In this context, a novel transmission scheme referred to as blind interference alignment (BIA) is proposed in [169] in order to achieve multiplexing gain without CSI at the transmitters. The main idea of BIA is based on exploiting the channel correlation among users, which are able to modify their radiation pattern based on the concept of reconfigurable antenna, during a period of time in which the physical channel does not vary. In [29], the concept of a reconfigurable photodetector is proposed in order to implement BIA for VLC systems. Basically, a reconfigurable photodetector consists of an ADR where the PDs are connected to a single signal processing chain and provide a linearly independent channel response each due to the angle diversity. Interestingly, it is shown that beyond the lack of need for CSI at the transmitters, BIA provides satisfactory data rates avoiding data sharing among optical APs, only requiring synchronization among them, therefore reducing the complexity of the backhaul. However, the received signal for BIA suffers a noise enhancement that is proportional to the number of users, and it requires a coherence period that is exponentially proportional with base the number of optical APs and exponent equal to the number of users. As a consequence, BIA results in being more useful for small VLC networks. As discussed in the following section, this issue makes the use of clustering strategies mandatory when applying BIA in large VLC networks.

Moreover, the main features of all these schemes are summarized in Table 4.

## 5. Precoding as Cooperative and Hybrid Networks

So far, we have focused on both cooperative and precoding schemes as a means of improving the interference management and the resilience to blocking effects. However, as the size of the VLC network increases, i.e., for a large number of optical APs and users, these schemes are subject to some drawbacks. Cooperative schemes require a large number of orthogonal resources, in order to avoid the inter-cell interference and, as the room size grows, the complexity involved by cooperating from multiple APs increases exponentially. On the other hand, precoding schemes typically assume two conditions; (i) full connectivity between all the optical APs to every user and (ii) linearly independent channel responses between transmitters and users. It can be easily checked that both conditions are affected by blocking effects, since obviously a blocked link means losing connectivity in the VLC network. However, mixing up these two ingredients, cooperative and precoding schemes, it is possible to generate a VLC network providing continuous high spectral efficiency in the whole scenario, good interference management and resilience to blocking effects.

### 5.1. Clustering for VLC. Intra-Cluster and Inter-Cluster Interference

**Figure 19:**
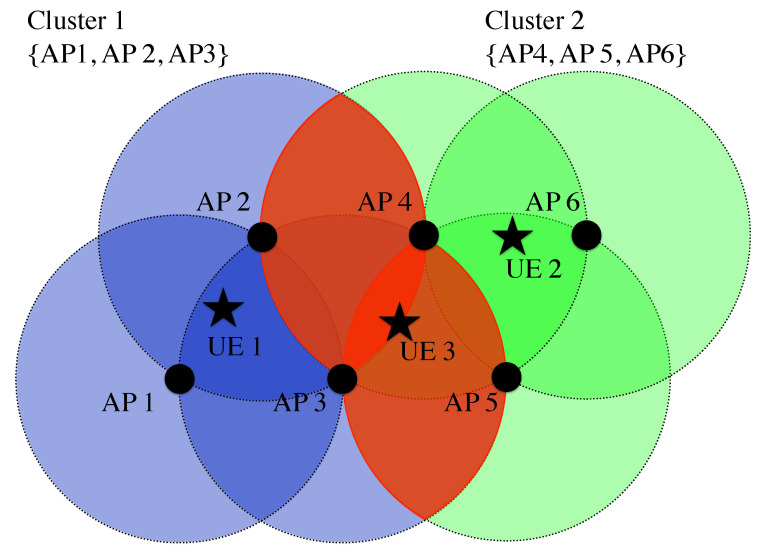
Network centric clustering. The clusters 1 and 2 are obtained by grouping the sets of optical APs {AP 1, AP 2, AP 3} and {AP 4, AP 5, AP 6} represented by blue and green colours, respectively. Red color represents the inter-cluster interference.

For RF systems, clustering is a well-known technique for both increasing the achievable DoF through precoding schemes and improving the spectral efficiency by reusing the frequency, i.e., a specific bandwidth, among the clusters [170,171]. Following a network-centric approach, the deployment of APs, e.g., base stations or attocells, is divided into clusters according to a predefined methodology, as is shown in Figure 19. Thus, in these multi footprint schemes, the intra-cluster interference is managed by implementing precoding schemes as described in Section 4. For instance, UE 1 and UE 2 in Figure 19 can be served simultaneously, i.e., avoiding orthogonal resource allocation such as TDMA, while minimizing or even cancelling the intra-cluster interference through any signal processing technique such as the cooperative or precoding schemes described in this work. However, UE 3 on the cluster edge may be subject to inter-cluster interference. In Section 3, the use of cooperative schemes such as FFR is proposed for managing the interference at the cell/cluster edge.

From the signal processing perspective, which can be applied to both RF and VLC systems, the fundamental limits of cooperation are analyzed in [88]. Theoretically, the spectral efficiency grows linearly as the transmitted power and the size of the network increase. In [88], it is demonstrated that this is not true in practice because of the need for pilot transmission associated to CSI and the constraints given by the capacity of the backhaul links, which are described in Section 4.1.2. As a consequence, cooperation is only possible within clusters of limited size, and therefore, it is required to manage both intra-cluster and inter-cluster interference. This way, the use of index coding by exploiting the network topology instead of CSI is proposed in [172].

Moving to VLC, each optical AP provides a small and confined area of coverage usually referred to as attocell. Therefore, VLC systems lay into the framework of the heterogeneous networks when they are considered as an element of the cellular network. In this sense, a comparison of the area spectral efficiency between femtocells and attocells composed of a single optical APs is carried out in [173]. It is shown that the area spectral efficiency achieved by VLC outperforms the performance of the femtocell system. Similarly, the energy efficiency, i.e., the costs in Joules for transmitting 1 bit, in VLC networks, is analyzed in [174].

As described in Section 3, several optical APs can be grouped to form an optical cell from a network centric perspective. Thus, the following question may be arisen; Are the well-known clustering strategies derived for RF suitable for VLC? The general answer is not due to the small and confined area of coverage of each optical APs. In the following, the concepts of user-centric clustering applied to VLC networks and hybrid RF/VLC networks are presented, taking into consideration the blocking effects that may appear in VLC networks.

### 5.2. User-Centric VLC Networks

The concept of user-centric clustering was firstly proposed in the framework of the heterogeneous networks. Specifically, in [175], a novel user-centric approach is proposed for CoMP dense cellular networks. Considering a multiple tier heterogeneous cellular network, the performance of user-centric clustering applied to the small cell tiers is analyzed in [176]. It is shown that the user-centric clustering approach outperforms the spectral efficiency achieved by the network-centric approaches. However, the works [175,176] are focused on heterogeneous networks following a Poisson point process. On the other hand, VLC systems do not interfere with other tiers, e.g., macro or micro cells, and the optical APs are typically distributed uniformly.

For VLC, the applications of user-centric clustering are firstly proposed in [35]. This work introduces the concept of an amorphous optical cell based on merging several optical APs cooperating among them so that the intra-cluster interference is managed by the considered cell. In [177], the authors present a novel methodology for generating amorphous optical cells for VLC, with the aim of improving the energy efficiency. It is worth noting that the authors of [177] combine OFDM transmission, cooperation among optical cells and precoding within each cell. Focusing on the intra-cluster and inter-cluster interference management, the formation of clusters from a user-centric perspective considering cooperation and precoding schemes is proposed in [106]. Those schemes can be considered multi footprint. The use of BIA schemes combined with user-centric clustering for VLC networks is proposed in [178,179]. It is shown that generating amorphous clusters with a limited number of optical APs and users reduces the demands on SNR and coherence time, which is given by the mobility of the users, and therefore, reducing the system requirements for implementing BIA.

**Figure 20:**
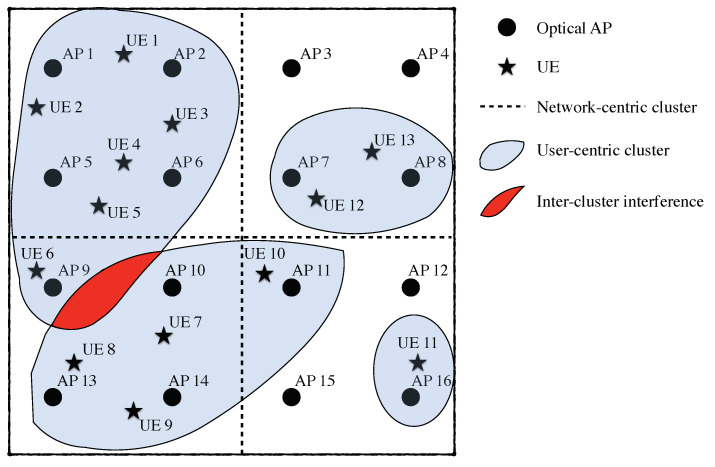
Example of user-centric cell formation.

For illustrative purposes, the cluster formation following a user-centric approach in a VLC network comprising L=16 optical APs is shown in Figure 20. First, notice that a network-centric approach forming 4 clusters comprising 4 optical APs each generates uniform cells that do not adapt to the distribution of the users. On the other hand, for the user-centric approach, the optical cells are formed in an elastic fashion, i.e., each cell corresponds to a cluster of optical APs that provides an amorphous coverage area that adapts to the user distribution. It can be seen that the user-centric approach minimizes the inter-cluster interference. However, it may appear and should be managed according to a specific strategy. At this point, it is worth recalling that any cooperative or precoding scheme can be implemented in order to manage the intra-cluster interference.

The formation of the user-centric clusters must consider the network topology, i.e., the position of the optical APs and the users. In [106], graph theory is applied to form these user-centric clusters [180]. The K-means algorithm is proposed as a means of generating clusters adapted to the distribution of the users in [178,179]. Moreover, the original K-means algorithm can be modified in order to satisfy constraints given by a specific transmission scheme, e.g., the limitations of BIA are considered in [178]. Focusing on the energy efficiency of the VLC networks using ZF precoding, a variation of the K-means algorithm is proposed in [181]. It is interesting to remark that the K-means algorithm is a primitive form of machine learning. At this point, notice that novel machine algorithms for clustering could be applied to VLC networks.

### 5.3. Hybrid RF/VLC Networks

Indoor VLC networks are usually deployed in scenarios where an RF communication system, e.g., WiFi, femtocells or other cellular network, are already available. Enabling cooperation among both systems leads to the concept of the hybrid VLC/RF network firstly proposed in [182]. In this sense, although VLC systems outperform the spectral efficiency of RF-based small cells [173], the cooperation between VLC and RF systems provides several benefits: enabling load balancing between VLC and RF networks, and this way, also overcoming VLC LoS-link blockages; guaranteeing users mobility by providing a seamless and robust coverage; and providing a comfortable RF uplink for VLC networks.

The design of VLC/RF networks taking into consideration the number of optical APs and served users is studied in [183]. In [184], dynamic load balancing in a hybrid VLC/RF network is proposed. It is shown that hybrid VLC/RF networks are useful for providing high data rates in areas where the received optical power is not high enough, i.e., shadowed areas. The load balancing algorithms for hybrid VLC/RF are improved in [103], considering both fairness and data rate in order to satisfy a network quality of service. Furthermore, considering the probability of blocking (shadowing), the performance of hybrid VLC/RF networks is analyzed in [102] using game theory. Interestingly, it is shown that there exists a trade-off between FoV, probability of blocking and network performance. This way, the implementation of BIA based on the concept of reconfigurable photodetector, which provides a wide FoV comprising several PDs, in hybrid VLC/RF networks is proposed in [179].

Beyond the application of load balancing algorithms for optimizing the performance of the network, hybrid VLC/RF systems can be considered for several purposes, and they are also classified into the multi footprint category.

## 6. Open Issues for Cooperative and Precoding Schemes in VLC

This work presented a comprehensive survey about the implementation of cooperative and precoding schemes in VLC systems are presented. However, there is still room for research in this field, and we have identified the following open issues:

### 6.1. Realistic Backhaul Links

Both cooperative and precoding schemes rely on an effective backhaul link for either inter-AP or AP-central controller connections. Traditionally, backhaul links have been assumed to be ideal [123], i.e., lossless, noiseless and instantaneous, that considers to provide an unlimited capacity. However, this is an unrealistic scenario, which makes cooperative and precoding schemes inaccurate. Considering a realistic backhaul link is essential for evaluating the overall performance of the system. As shown in Section 2.1.3, several backhaul techniques have been proposed in the literature, considering both wired and wireless solutions. However, there is still a lack of comprehensive studies of backhaul link in terms of cost-efficient, energy-efficient, spectrum-efficient, when large scenarios containing tens and hundreds of APs are deployed. Indeed, this is considered as an open issue in the IEEE 802.11bb VLC standard [17]. As a consequence, the analysis of cooperative and precoding schemes in a multi-cell scenario when considering realistic backhaul link is also an open issue that should be addressed in the near future.

### 6.2. Angle Diversity Receivers for Overcoming the Shadowing and Blocking Effects

VLC are not subject to small-scale effects that are common for RF communications. This issue may lead to correlated channel responses among users, and therefore, to obtain a poor performance when applying precoding schemes that cancel or minimize the interference among users. In [161], receivers composed of multiple PDs with distinct FoVs are proposed for minimizing the correlation among the channels provided by the PDs of the same user. Similarly, ADRs may provide enough channel diversity to generate uncorrelated channel responses [76,158]. Motivated by [135], the use of ADRs can also be managed as multi-user diversity, which may provide a performance close to the asymptotic capacity for ZF precoding. This way, in [78], the concept of vROA photodetector is proposed for modifying the orientation angle of each user in order to provide a large channel diversity that maximizes the performance of ZF precoding. However, the use of ADRs or vROA photodetectors for cooperative/precoding schemes with the aim of overcoming the shadowing and blocking effects in VLC is still an open issue that requires one to develop novel ideas and algorithms.

### 6.3. Is the Concept of Massive Mimo Applicable to VLC?

Massive MIMO has been widely proposed for improving the achievable rate in RF communications [185]. Let us consider the channel matrix described in (22). The key idea of massive MIMO is to increase the number of transmit antennas so that the channel responses of the users are asymptotically orthogonal. That is, the channel responses among users are orthogonal, since the channel of each user comprises a large number of dimensions (transmitters) in comparison with the total number of users. As a consequence, linear precoding schemes, subject to a low complexity in comparison with DPC, obtain a performance close to the capacity. Mathematically, this condition is given by

(24) $$\lim_{M\to \infty }=\bar{H}{\bar{H}}^{H}=MI_{K},$$

which holds if the entries of $\bar{H}$ (see (22)) are independent and identically distributed. For RF systems, this condition is naturally satisfied, assuming rich-scattering environments. Thus, simple linear precoding such as ZF provides a performance close to the capacity [185]. However, due to the deterministic behaviour of the optical channel (see (16)), the resulting channel matrix is typically ill-conditioned to exploit the benefits of massive MIMO in VLC systems. In [186], an alternative precoding based on single value decomposition is proposed to overcome the high correlation of the ill-conditioned channel matrix. The channel estimation in a VLC system, which requires FDD operating mode, is analyzed in [187] when the number of optical APs grows enough to consider the massive MIMO condition. Focusing on indoor positioning, the use of large-scale VLC systems is considered in [188]. However, there are still several issues to analyze about the implementation of massive MIMO in VLC, such as the impact of ADRs or vROA photodetectors, the capacity bounds as the number of optical APs grows or the precoding schemes that achieve rates close to the capacity.

### 6.4. User-Centric Approaches for Precoding Schemes

The implementation of precoding schemes in VLC systems typically assumes full connectivity between the optical APs and users. However, in practical scenarios, only the signals from some optical APs are received at each user, due to the small and confined area of coverage provided by each optical AP. Moreover, the received signal may be subject to blocking effects. In this context, traditional clustering from a network-centric point of view is usually useless since it does not consider the distribution of the users [189]. Recently, the user-centric approach has been proposed as a means of achieving optimal clustering in VLC systems [35,42]. Nevertheless, depending on the resulting clusters, the task of designing a transmission strategy based on either FR or precoding schemes is not straightforward [189]. Specifically, in [106], an alternative scheduling is derived for the use of ZF precoding under user-centric clustering. However, novel scheduling algorithms and precoding schemes are still required to exploit the paradigm introduced by the user-centric approaches in VLC systems.

### 6.5. CSI Prediction for Mobility

The deployment of LED lamps combined with VLC can be considered for providing indoor positioning [190]. This fact allows us to track the position of the users, and therefore, to predict their CSI, which recalls its deterministic behaviour according to (16), as the users may move onto the scenario [191]. Considering the mobility of the users, the impact of shadowing (LoS-link blocking) in a tracking VLC system is analyzed in [192]. However, to the best or our knowledge, the use of tracking techniques improving the cooperation of precoding techniques in VLC is currently an open issue. Moreover, ADRs and vROA photodetectors offer a new paradigm in order to design tracking schemes that allow us to create novel cooperative and precoding transmission techniques.

## 7. Conclusions

In this paper, a comprehensive survey was presented for cooperative and precoding schemes in indoor VLC systems. VLC is an enabling technology for beyond 5G services. One of its key limitations is the likely LoS-link blockage and the inter-cell interference when deploying multi- and small- cell scenarios. In this regard, we first identified the architecture, system model and typical scenarios for indoor VLC. Subsequently, the different cooperative and precoding schemes for mitigating the LoS-link blockage and the inter-cell interference presented in the literature were discussed, highlighting the pros and cons of each of them. Precoding schemes that serve as cooperative for overcoming LoS-link blockages were also classified. This survey also identified and classified the state-of-the-art works depending on its functionality: interference mitigation, LoS-link blockage avoidance, and additionally, their complexity and requirements were evaluated. To push the research in cooperative and precoding schemes for VLC further, open issues in this domain were finally detailed.

## Figures and Tables

**Table 1:** List of acronyms in this survey.

ACO-OFDM	Asymmetrically clipping optical–orthogonal frequency division multiplexing
ADO-OFDM	Asymmetrically clipped direct current biased optical OFDM
ADR	Angle diversity receiver
AP	Access point
AWGN	Additive white Gaussian noise
BD	Block diagonalization
BER	Bit error rate
CSI	Channel state information
CU	Central unit
DCO-OFDM	DC-biased optical-orthogonal frequency division multiplexing
DD	Direct detection
DPC	Dirty paper coding
FDD	Frequency division duplex
FFR	Fractional frequency reuse
FFT	Fast Fourier transform
FoV	Field of view
FR	Frequency reuse
IFFT	Inverse fast Fourier transform
IM	Intensity modulation
IR	Infrared
JT-CoMP	Joint transmission—Coordinated multipoint
LED	Light emitting diodes
LoS	Line-of-sight
LTE	Long Term Evolution
MIMO	Multiple-input multiple-output
MMSE	Minimum mean squared error
M-PAM	Multi-level pulse amplitude modulation
M-PPM	Multi-level pulse position modulation
MRC	Maximum ratio combining
MSE	Mean squared error
NLoS	Non-line-of-sight
NOMA	Non-orthogonal multiple access
OOK	On-off keying
O-OFDM	Optical orthogonal frequency division multiplexing
PD	Photodiode
PLC	Power line communication
PWM	Pulse width modulation
QAM	Quadrature amplitude modulation
RGB	Red-green-blue
RF	Radio frequency
SDMA	Spatial division multiple access
SINR	Signal-to-interference-plus-noise ratio
SISO	Single-input-single-output
SNR	Signal-to-noise ratio
TDD	Time division duplex
TDMA	Time division multiple access
VLC	Visible light communications
ZF	Zero forcing

**Table 2:** List of variables in this survey.

*N*	Number of sub-carriers
$P_{max}$	Maximum Optical Power
$I_{H}$	Higher level current
Δfm	Measurement bandwidth
$G\left(\Psi_{c}\right)$	Gain of hemispherical concentrator
$\Psi_{c}$	Field of View
$\frac{\Delta \lambda }{2}$	Spectral half-power bandwidth
$\lambda \left(\varphi ;\hat{\varphi }\right)$	Shifting to shorter wavelength at non normal incidences
$n_{i}$	Effective index of the input layer
*K*	Number of users
$E_{i}$	Incident energy in W/cm${}^{2}$
$y^{\left[k\right]}\left(t\right)$	Received signal by user *k* at time *t*
$h^{\left[k\right]}\left(t\right)$	Channel response vector between the *M*-th optical transmitter and user *k* at time *t*
$h_{LOS,m}^{\left[k\right]}$	LoS channel component from optical AP *m*-th to user *k*
$h_{fe}\left(t\right)$	Time-domain CIR for the front-end
X[n]	Frequency-domain data in *n*-th sub-carrier
$H^{\mathrm{SR}}$	Reflection-based channel
$H_{\mathrm{NLoS}}^{\mathrm{SD}}$	Channel from source to destination (NLoS component)
$H_{\mathrm{NLoS}}^{\mathrm{RD}}$	Channel from relay to destination (NLoS component)
*t*	Time instant
$\varphi_{m}^{\left[k\right]}$	Incident angle for user *k* from AP *m*
γ	Responsivity of PD
$\rho_{1}$	Reflectivity of the main region
$\sigma_{z}^{2}$	Variance of Gaussian noise
|F|	Number of frequency sub-bands per color
*Q*	Cluster size
$w^{\left[k\right]}$	Precoding vector associated to user *k*
$\theta_{ep}$	Resources allocated for channel estimation
$\theta_{fb}$	Resources allocated for feedback
$L_{CP}$	Number of samples of Cyclic Prefix
$I_{L}$	Lower level current
$A_{PD}$	Area of detection of PD
$T_{\phi }$	Time for peak transmission
φ	Generic angle of incidence
$\lambda_{0}$	Wavelength of transmitted signal
υ	Lambertian radiation index
$n_{s}$	Effective index of the sparcer layer
*M*	Number of Access Points
$C^{\left[k\right]}$	Lower bound capacity for user *k*
$d_{\left[m\right]}^{\left[k\right]}$	Distance between *m*-th AP and user *k*
$h_{m}^{\left[k\right]}\left(t\right)$	Channel response for user *k* from Access Point *m* at instant *t*
$h_{diff}$	Contribution of the nLoS components
$h_{fs}\left(t\right)$	Time-domain free-space channel component
$H_{fe}$	Frequency-domain CIR for the front-end
${\hat{n}}^{\left[k,p\right]}$	Orientation vector of p-th PD of user k-th
$H_{\mathrm{LoS}}^{\mathrm{SD}}$	Channel from source to destination (LoS component)
$H_{\mathrm{LoS}}^{\mathrm{RD}}$	Channel from relay to destination (LoS component)
$s^{\left[k\right]}$	Time-domain symbol to user *k*
$\phi_{m}^{\left[k\right]}$	Irradiance angle for user *k* from AP *m*
$\phi_{1/2}$	Radiation semi-angle
$A_{\mathrm{room}}$	Area of the room
ρ	Average reflectivity
|C|	Number of chip colors
|G|	Number of resources
|S|	Number of sector per cell
*P*	Number of orthogonal Pilots
$\theta_{cd}$	Resources allocated for coherence detection
$T_{S}$	Symbol Period

**Table 3:** VLC cooperative techniques.

Technique	Interference Mitigation	LoS-Link Blockage Avoidance	Complexity	Source
FFR	Yes	No	Low	[109,110]
JT-CoMP	Yes	Yes	High	[117,118,119,120,121]
Pre-defined JT-CoMP	Yes	Yes	Low	[31,46,104,111]
Relay-based	No	Yes	High	[45,125,126,127]
NOMA	Yes	No	Medium	[48,105,128,129]
SDMA	Yes	No	Medium	[130,131,132]

**Table 4:** VLC precoding techniques.

Technique	CSI Requirements	Interference Mitigation	LoS-Link BLOCKAGE Avoidance	Complexity	Source
OMIMO	Receiver	No	No	Low	[32,154,155]
DPC	Transmitters and Receivers	Yes	Medium	Very High	[160]
Linear ZF	Transmitters	Yes	Medium	High	[161,162,167]
BD	Transmitters and Receiver	Yes	High (ADRs)	High	[167]
max-min SINR	Transmitter	Yes	Medium	Medium	[76,166]
MMSE	Transmitter	Yes	Medium	High	[163]
CoMP-MMSE	Transmitters	Yes	High	Medium	[118]
BIA	Receiver	Yes	Medium (ADRs)	Medium	[29]
